# Functional validation of putative toxin-antitoxin genes from the Gram-positive pathogen *Streptococcus pneumoniae*: *phd-doc* is the fourth *bona-fide* operon

**DOI:** 10.3389/fmicb.2014.00677

**Published:** 2014-12-05

**Authors:** Wai Ting Chan, Chew Chieng Yeo, Ewa Sadowy, Manuel Espinosa

**Affiliations:** ^1^Molecular Microbiology and Infection Biology, Centro de Investigaciones Biológicas, Consejo Superior de Investigaciones CientíficasMadrid, Spain; ^2^Faculty of Medicine and Health Sciences, Universiti Sultan Zainal Abidin, Kuala TerengganuTerengganu, Malaysia; ^3^Department of Molecular Microbiology, National Medicines InstituteWarsaw, Poland

**Keywords:** *Streptococcus pneumoniae*, pneumococcal toxin-antitoxin loci, Phd-Doc, Green Fluorescent Protein, self-regulation

## Abstract

Bacterial toxin-antitoxin (TAs) loci usually consist of two genes organized as an operon, where their products are bound together and inert under normal conditions. However, under stressful circumstances the antitoxin, which is more labile, will be degraded more rapidly, thereby unleashing its cognate toxin to act on the cell. This, in turn, causes cell stasis or cell death, depending on the type of TAs and/or time of toxin exposure. Previously based on *in silico* analyses, we proposed that *Streptococcus pneumoniae*, a pathogenic Gram-positive bacterium, may harbor between 4 and 10 putative TA loci depending on the strains. Here we have chosen the pneumococcal strain Hungary^19A^-6 which contains all possible 10 TA loci. In addition to the three well-characterized operons, namely *relBE2, yefM-yoeB*, and *pezAT*, we show here the functionality of a fourth operon that encodes the pneumococcal equivalent of the *phd-doc* TA. Transcriptional fusions with gene encoding Green Fluorescent Protein showed that the promoter was slightly repressed by the Phd antitoxin, and exhibited almost background values when both Phd-Doc were expressed together. These findings demonstrate that *phd-doc* shows the negative self-regulatory features typical for an authentic TA. Further, we also show that the previously proposed TAs XreA-Ant and Bro-XreB, although they exhibit a genetic organization resembling those of typical TAs, did not appear to confer a functional behavior corresponding to *bona fide* TAs. In addition, we have also discovered new interesting bioinformatics results for the known pneumococcal TAs RelBE2 and PezAT. A global analysis of the four identified toxins-antitoxins in the pneumococcal genomes (PezAT, RelBE2, YefM-YoeB, and Phd-Doc) showed that RelBE2 and Phd-Doc are the most conserved ones. Further, there was good correlation among TA types, clonal complexes and sequence types in the 48 pneumococcal strains analyzed.

## Introduction

Toxins-antitoxins (TAs) were discovered in the early 1980s and, later on, their cellular function was extensively studied especially in the past two decades. However, until now the world of TAs is still perplexing. TAs are found abundantly in prokaryotes, especially in free-living bacteria, as well as in archaea, and probably in fungi but so far, not in other eukaryotes (Pandey and Gerdes, 2005). In general, a TA locus consists of two genes organized as an operon, with the antitoxin gene, which is generally precedes the toxin gene, encoding either an untranslated RNA or a labile protein that neutralizes the toxicity of its cognate toxin, whereas the toxin gene encodes a toxin protein that binds to the cellular targets and halts essential cell processes. Under certain circumstances such as stress, the system would be triggered by a rapid degradation of the antitoxin, thus liberating the toxin to act on its target. Most known toxins function as RNases (Christensen et al., 2001; Nariya and Inouye, 2008; Jørgensen et al., 2009; Yamaguchi and Inouye, 2009), whereas other toxins target essential cellular components such as DNA gyrase (Van Melderen, 2002), cell wall (Mutschler et al., 2011), and EF-Tu elongation factor (Castro-Roa et al., 2013). TAs have been classified into five types, depending on how the antitoxin counteracts the toxin. Type II TAs, in which the antitoxin is a protein that binds avidly to the toxin protein to form an inert complex, are the most widely studied (Makarova et al., 2009; Leplae et al., 2011). When found on plasmids, TAs function primarily in the stable maintenance of the plasmid, post-segregationally killing off any plasmid-free daughter cells that developed. The functions of the chromosomally-encoded bacterial TAs are still intriguing. They might not be essential, but they have been reported to be involved in a variety of cellular processes related to global stress responses (Christensen et al., 2001), programmed cell death (Engelberg-Kulka and Glaser, 1999), maintenance of mobilomes (Rowe-Magnus et al., 2003; Szekeres et al., 2007), persistence (Gerdes and Maisonneuve, 2012), biofilm formation (Harrison et al., 2009; Soo and Wood, 2013), niche colonization (Norton and Mulvey, 2012), virulence (Ren et al., 2012), phage abortive infection system (Fineran et al., 2009; Dy et al., 2014), and other important cellular processes (Goeders and Van Melderen, 2014). Some bacterial genomes are bountiful in TAs, e.g., Mycobacterium tuberculosis encodes 30 functional TAs (Ramage et al., 2009), which makes their intricacy bewildering. The ability of TAs to impart growth control may attribute to the slow growth and dormant state, which are the hallmarks of latent tuberculosis infection (Schifano and Woychik, 2014), but the mechanisms involved, and how many TAs may be responsible for these phenotypic traits are still unknown. It is still unclear whether the TAs within the same host could cross-talk. However, some studies have shown that expression of one TA might activate another (Garcia-Pino et al., 2008). Whether numerous TAs would provide cumulative or synergistic effect to the cells is something worth to ponder.

*Streptococcus pneumoniae* (the pneumococcus) is a Gram-positive bacterium, pathogenic for humans and responsible for both infections in respiratory tracts as well as invasive infections, and associated with significant morbidity and mortality (Chan et al., 2012). Furthermore, clinical isolates of pneumococci show a high degree of variability, probably due to the recombinogenic nature of this bacterium (Claverys et al., 2006; Baquero, 2009). We had previously identified up to 10 putative Type II pneumococcal TAs based on *in silico* data mining (Chan et al., 2012), and three of them had already been proven to be functional, namely *relBE2*, *yefM-yoeB*, and *pezAT* (Chan et al., 2013). Here, we have undertaken the validation and characterization of the rest of the putative pneumococcal TAs. We show that *S. pneumoniae* encodes a fourth functional TA operon, *phd-doc*, which corresponds to a *bona fide* TA. Further, we experimentally ruled out some of the predicted pneumococcal gene pairs as typical TAs, since some of these putative toxins turned out to be either non-toxic, or possibly toxic but lacking a cognate antitoxin gene.

## Materials and methods

### Bacterial strains and growth conditions

*S. pneumoniae* R6 (Hoskins et al., 2001) and *Escherichia coli* Top10 [F¯*mcrA*Δ(*mrr-hsd*RMS*-mcrBC*) Φ 80*lacZ*ΔM15 Δ*lacX74 recA1 araD139* Δ(*ara*A*leu*)*7697 galUgalKrpsL*(Str^r^) *endA1 nupG*] were used as homologous and heterologous hosts for verification of putative TAs from *S. pneumonia* Hungary^19A^-6 (McGee et al., 2001) *via* overexpression assays. *S. pneumoniae* R6 was grown in AGCH medium (Lacks, 1968) with 0.3 sucrose and 0.2% yeast extract at 37°C in static water bath, and supplemented with erythromycin (1 μg/ml) or tetracycline (1 μg/ml) when the cells harbored recombinant plasmids pLS1ROM-MCS (Lacks et al., 1986; Ruiz-Masó et al., 2012), or pAST (Ruiz-Cruz et al., 2010), respectively. *E. coli* cultures were grown in TY medium (Sambrook and Russel, 2001), at 37°C with agitation at 250 rpm (for liquid medium), and supplemented with kanamycin (50 μg/ml) when the cells harbored plasmid pFUS2 (Lemonnier et al., 2003) or its derivatives.

### DNA manipulations, sequencing, and sequence data analysis

DNA manipulations and other molecular biology techniques were done following standard protocols (Sambrook and Russel, 2001). *S. pneumoniae* genomic DNA was extracted using the Bacterial Genomic DNA Isolation Kit (Norgen Biotech Corp.). Plasmid DNA from both *S. pneumoniae* and *E. coli* was isolated using the High Pure Plasmid Isolation kit (Roche). However, for *S. pneumoniae*, the plasmid extraction protocols were slightly modified as described (Ruiz-Cruz et al., 2010). DNA fragments and PCR products were purified with the NZYGelpure from NZYTech. DNA was sent for automated Sanger sequencing in Secugen S.L., Centro de Investigaciones Biológicas, CSIC, Madrid. DNA sequences were analyzed using the BioEdit Sequence Alignment Editor version 7.0.4.1 (Hall, 1999).

### Construction of recombinant plasmids with *S. pneumoniae* hungary^19A^-6 putative TAs

All plasmids and primers used in this study are shown in Table 1, and the details are described below.

**Table 1:** **Recombinant plasmids used in this study**.

**Plasmids**	**Descriptions and primers used**
pFUS2, (Lemonnier et al., 2003)	A 4.5 kb vector with ori-pBR322 which harbors an arabinose-inducible P_BAD_ promoter upstream of a multiple cloning site
pFUS2SD	An SD consensus ′AGGAGG′ was inserted downstream of P_BAD_ promoter and upstream of EcoRI restriction site of pFUS2 plasmid. This recombinant plasmid was used for the cloning of putative toxins from *S. pneumoniae* Hungary^19A^-6 and subsequently for overexpression assays in *E. coli* Top10
	Forward primer: 5′-GGGCTAGCGAATTCGAGCTC-3′
	Reverse primer: 5′-GATCGAATTCCCTCCTGCTAGCCCAAAAAAACGG-3′
pFUS2SD_YoeB	pFUS2SD with *yoeB* from *S. pneumoniae* R6; as positive control in overexpression assays in *E. coli* Top10
	Forward primer: 5′-GATAGAATTCATGCTACTCAAGTTTACAGA-3′
	Reverse primer: 5′-GACAAAGCTTTTAGTAATGATCTTTAAAGG-3′
pFUS2SD_RelE1	pFUS2SD with *relE1* from *S. pneumoniae* Hungary^19A^-6
	Forward primer: 5′-AGTAGAATTCGTGCTTAAGATTCGTTATCA-3′
	Reverse primer: 5′-TCATAAGCTTTTAAAATAAATCACTGTGAC-3′
pFUS2SD_HicA	pFUS2SD with *hicA* from *S. pneumoniae* Hungary^19A^-6
	Forward primer: 5′-AGTAGAATTCATGCCTATGACACAAAAAGA-3′
	Reverse primer: 5′-TCATAAGCTTGCAGGGGCTGAATTTTACAA-3′
pFUS2SD_COG2856CA	pFUS2SD with COG2856CA gene from *S. pneumoniae* Hungary^19A^-6
	Forward primer: 5′-AGTAGAATTCATGGATTTAAGTAATAAAGC-3′
	Reverse primer: 5′-TTGTAAGCTTTTATCCTAATGACATTTCCT-3′
pFUS2SD_COG2856B	pFUS2SD with COG2856B gene from *S. pneumoniae* Hungary^19A^-6
	Forward primer: 5′-AGTAGAATTCATGACTGAAAAAGAATTTTC-3′
	Reverse primer: 5′-TCATAAGCTTTTAATTAAGTAGTGCTAAAT-3′
pFUS2SD_Ant	pFUS2SD with *ant* from *S. pneumoniae* Hungary^19A^-6
	Forward primer: 5′-AGTAGAATTCATGAACGAACTCATCAACGT-3′
	Reverse primer: 5′-TCATAAGCTTCTAATTAAGGAACTTATCGA-3′
pFUS2SD_Bro	pFUS2SD with *bro* from *S. pneumoniae* Hungary^19A^-6
	Forward primer: 5′-AGTAGAATTCATGAACGAAATTTTTAATTT-3′
	Reverse primer: 5′-TCATAAGCTTCTACCCTTCGTCAAATGAGT-3′
pLS1ROM-MCS, (Lacks et al., 1986; Ruiz-Masó et al., 2012)	A 6.8 k vector derived from plasmid pLS1 that harbors a maltose-inducible P_M_ promoter upstream of a multiple cloning site. This plasmid was used for the cloning of putative TAs from *S. pneumoniae* Hungary^19A^-6 and subsequently for overexpression assays in *S. pneumoniae* R6
pLS1ROM_HicA	pLS1ROM-MCS with *xreA* from *S. pneumoniae* Hungary^19A^-6
	Forward primer: 5′-AGTAAAGCTTGGTGTTGTCAGGAGGTAAAA-3′
	Reverse primer: 5′-AGTCACTAGTGAATTTTACAACCCAGCTTG-3′
pLS1ROM_RelE1	pLS1ROM-MCS with *xreA* from *S. pneumoniae* Hungary^19A^-6
	Forward primer: 5′-GTAGAAGCTTATTTGATGGAGGACTTACG-3′
	Reverse primer: 5′-AGTCACTAGTGAGAACCCCCTTAAAATAGA-3′
pLS1ROM_COG2856CA	pLS1ROM-MCS with *xreA* from *S. pneumoniae* Hungary^19A^-6
	Forward primer: 5′-GTAGAAGCTTGGAAAAAGGTGAAAGGAATA-3′
	Reverse primer: 5′-AGTCACTAGTTTCCTCATACTTCCCTTGCG-3′
pLS1ROM_COG2856B	pLS1ROM-MCS with *xreA* from *S. pneumoniae* Hungary^19A^-6
	Forward primer: 5′-GTACAAGCTTAGAAAGCAATAATAATAAAA-3′
	Reverse primer: 5′-AGTCACTAGTTTTTCTCTCCTTTTTTAATT-3′
pLS1ROM_XreA	pLS1ROM-MCS with *xreA* from *S. pneumoniae* Hungary^19A^-6
	Forward primer: 5′-GATCCCAAGCTTCTATTATTTGTCAATGAATA-3′
	Reverse primer: 5′-GATCCCACTAGTTTATTCCTCCAGCAATTGTT-3′
pLS1ROM_Ant	pLS1ROM-MCS with *ant* from *S. pneumoniae* Hungary^19A^-6
	Forward primer: 5′-GATCCCAAGCTTTTCAATTGCAAATTATTTAA-3′
	Reverse primer: 5′-GATCCCACTAGTCTAATTAAGGAACTTATCGA-3′
pLS1ROM_XreA-Ant	pLS1ROM-MCS with *xreA-ant* from *S. pneumoniae* Hungary^19A^-6
	Forward primer: 5′-GATCCCAAGCTTCTATTATTTGTCAATGAATA-3′
	Reverse primer: 5′-GATCCCACTAGTCTAATTAAGGAACTTATCGA-3′
pLS1ROM_XreB	pLS1ROM-MCS with *xreB* from *S. pneumoniae* Hungary^19A^-6
	Forward primer: 5′-GATCCCAAGCTTAGCACTAATACCAAGATGAA-3′
	Reverse primer: 5′-GATCCCACTAGTTTATATTTGATTAGAGAGCC-3′
pLS1ROM_Bro	pLS1ROM-MCS with *bro* from *S. pneumoniae* Hungary^19A^-6
	Forward primer: 5′-GATCCCAAGCTTACACGATTATTCACCCCTTG-3′
	Reverse primer: 5′-GATCCCACTAGTCTACCCTTCGTCAAATGAGT-3′
pLS1ROM_Bro-XreB	pLS1ROM-MCS with *bro-xreB* from *S. pneumoniae* Hungary^19A^-6
	Forward primer: 5′-GATCCCAAGCTTACACGATTATTCACCCCTTG-3′
	Reverse primer: 5′-GATCCCACTAGTTTATATTTGATTAGAGAGCC-3′
pLS1ROM_Phd	pLS1ROM-MCS with *phd* from *S. pneumoniae* Hungary^19A^-6
	Forward primer: 5′-GAGCGGCCGCAGGAGGAAATCAGATGGTAGTAAAAACAAGAAA-3′
	Reverse primer: 5′-CAGCGGATCCTCATTTTTCCACCAAAGCTT-3′
pLS1ROM_Doc	pLS1ROM-MCS with *doc* from *S. pneumoniae* Hungary^19A^-6
	Forward primer: 5′-GAGCGGCCGCAGGAGGAAATCAGATGACAATCTATTTGACAGA-3′
	Reverse primer: 5′-CAGCGGATCCTTACTTTTTGACCTTTTCTC-3′
pLS1ROM_Phd-Doc	pLS1ROM-MCS with *phd-doc* from *S. pneumoniae* Hungary^19A^-6
	Forward primer: 5′-GAGCGGCCGCAGGAGGAAATCAGATGGTAGTAAAAACAAGAAA-3′
	Reverse primer: 5′-CAGCGGATCCTTACTTTTTGACCTTTTCTC-3′
pAST, (Ruiz-Cruz et al., 2010)	A 5.5 kb vector derived from plasmid pMV158 that harbors a multiple cloning site between the *E. coli T1T2rrnB* terminator region and the promoter-less *gfp* gene
pAST_P	A 231 bp of DNA encompassing the putative promoter of *phd-doc* from *S. pneumoniae* Hungary^19A^-6 was inserted upstream of the *gfp* gene of pAST plasmid.
	Forward primer: 5′-CAGAGGATCCCTGTTGGAAATGTAGCGTAC-3′
	Reverse primer: 5′-CAGCGAGCTCCTGATTTCCTCCTTTGAAGT-3′
pAST_PPhd	A 471 bp of DNA encompassing the putative promoter of *phd-doc* and *phd* gene from *S. pneumoniae* Hungary^19A^-6 was inserted upstream of the *gfp* gene of pAST plasmid.
	Forward primer: 5′-CAGAGGATCCCTGTTGGAAATGTAGCGTAC-3′
	Reverse primer: 5′-CAGCGAGCTCTTGTCATTTTTCCACCAAAG-3′
pAST_PPhd	An 878 bp of DNA encompassing the putative promoter of *phd-doc* and *phd-doc* genes from *S. pneumoniae* Hungary^19A^-6 was inserted upstream of the *gfp* gene of pAST plasmid.
	Forward primer: 5′-CAGAGGATCCCTGTTGGAAATGTAGCGTAC-3′
	Reverse primer: 5′-CAGCGAGCTCTTACTTTTTGACCTTTTCTC-3′

*The underlined sequences were the restriction sites used for cloning*.

#### pLS1ROM-MCS derivatives

Seven putative pneumococcal TA genes from strain Hungary^19A^-6 (GI: 169832377) (Chan et al., 2012) were verified in the *S. pneumoniae* homologous host. Plasmid pLS1ROM-MCS (Lacks et al., 1986; Ruiz-Masó et al., 2012), which was derived from the streptococcal promiscuous plasmid pMV158 (Espinosa, 2013), harbors a tight maltose-inducible promoter P_M_. The pneumococcal putative toxin genes along with their Shine-Dalgano (SD) sequences were cloned downstream of promoter P_M_, and then transformed (Lacks, 1968) into *S. pneumoniae* R6 for overexpression assays. The seven putative toxin genes were: *relE1* (GI: 169832900), *hicA* (GI: 169834067), genes COG2856CA (GI: 169832377 and 169833989), gene COG2856B (GI: 169832735), *doc* (GI: 169833045), *ant* (*antirepressor*) (GI: 169833276; previously known as *bro1*, Chan et al., 2012) and *bro* (*baculovirus repeated orfs*) (GI: 169834001; previously known as *bro2*, Chan et al., 2012). In the case of the *doc* gene, its SD sequences were not apparent and thus we decided to use the SD sequences of its cognate antitoxin *phd*. Consequently, those which exhibited toxicity to the cells, i.e., *doc*, *ant* and *bro*, were further analyzed by cloning their cognate putative antitoxins *phd* (GI: 169832994), *xreA* (GI: 169832832; previously known as *xre*, Chan et al., 2012) and *xreB* (GI: 169834410; previously known as *xre*, Chan et al., 2012), as well as both putative TA operons along with their SD, respectively, into pLS1ROM-MCS, and then subjected to overexpression assays.

#### pFUS2SD and derivatives

The arabinose-inducible plasmid pFUS2 (Lemonnier et al., 2003) was used to construct recombinant plasmids with the seven putative toxins from *S. pneumoniae* Hungary^19A^-6 (as described above) to validate their functionality in *E. coli* heterologous host. Since TA operons are usually co-transcribed and some of the toxin genes which are located downstream of the antitoxin genes do not have an obvious SD sequence, we had constructed plasmid pFUS2SD (this study) by inserting an SD consensus sequence (AGGAGG) downstream of the arabinose-inducible P_BAD_ promoter of pFUS2 by reverse PCR and then self-ligation. This recombinant plasmid was subsequently used for the cloning of the seven putative toxin genes from Hungary^19A^-6 strain. In addition, the known toxin *yoeB* (GI: 15903627) from *S. pneumoniae* R6 was also constructed in pFUS2SD to serve as a positive control (Chan et al., 2011). All these genes were inserted at the EcoRI restriction site downstream of the SD sequence, and thus the start codons of all these genes were 6 bp apart from the SD sequence. The recombinant plasmids were transformed into *E. coli* Top10 (Sambrook and Russel, 2001) and used to conduct overexpression assays.

#### pAST derivatives

To verify the promoter activities and to characterize the transcriptional regulation of the promoter by pneumococcal Phd and Phd-Doc, transcriptional fusions were constructed to measure Green Fluorescent Protein (GFP) fluorescence. Plasmid pAST (Ruiz-Cruz et al., 2010), which harbors a promoter-less *gfp* gene was used for this study. The putative promoter of the *phd-doc* operon, the *phd* gene along with its promoter and the *phd-doc* genes along with their promoter were inserted upstream of the *gfp* gene of pAST plasmid, respectively. All the recombinant plasmids were transformed into *S. pneumoniae* R6 and then to evaluate the promoter activities by measuring GFP fluorescence.

### Overexpression assays of putative TAs in *S. pneumoniae* and *E. coli*

Cell cultures of *S. pneumoniae* R6 harboring the different recombinant plasmids were grown in AGCH medium supplemented with 0.3 sucrose and 0.2% yeast extract (Lacks et al., 1986) until optical density (OD)_650_ ~ 0.3 and subsequently subcultured to OD_650_ of ~0.02 in the same medium but with addition of 0.2% maltose to induce promoter P_M_. OD of the cultures was measured every hour for 8 h. *E. coli* Top10 with different recombinant plasmids were inoculated in TY medium (Maniatis et al., 1982) supplemented with 0.4% glucose to repress expression from the P_BAD_ promoter, and the cells were allowed to grow until OD_600_ of ~0.3. Cells were harvested and washed twice with TY medium, followed by suspension in TY medium to OD_600_ of ~0.02, and then induced with 0.4% arabinose. The assays were repeated three times and the mean values and the standard errors were calculated.

### GFP fluorescence assays

To characterize the promoter activities of pneumococcal *phd-doc*, fluorescence assays were conducted as previously described (Ruiz-Cruz et al., 2010). Briefly, *S. pneumoniae* carrying recombinant plasmids with various *gfp*-fusions were grown until OD_650_ ~ 0.3. Various volumes of cells (25 μl to 1 ml) were harvested and resuspended in 200 μl 1 × PBS buffer in a 96-well black plate, respectively. Fluorescence was measured with a Varioskan Flash Spectral Scanning Multimode Reader (Thermoscientific) by excitation at 488 nm with a bandwidth of 12 nm and detection of emission at 515 nm. Three independent cultures were analyzed and results of 1 ml of cultures were chosen to show in this manuscript. The cells were also subsequently used for microscopy examination.

### Cell morphology examination with phase-contrast and fluorescence microscopy

Cells from overexpression assays were harvested at 4 h and 8 h after induction. Cells were washed twice with 1 × PBS, resuspended in 4% paraformaldehyde and fixed at room temperature for 30 min. Cells were again washed once with 1 × PBS buffer and resuspended in the same buffer. The fixed cells were placed on polylysine coated microscope slides, air-dried and mounted with Vectashield (Vector Laboratories) before being covered with cover slides, and sealed with nail polish. The cells were examined under microscope (Olympus CKX41), and pictures were taken at 100 × magnification. To examine pneumococcal cells harboring various *gpf*-fused promoter-*phd-doc* recombinant plasmids, 10 μl of cells in 1 × PBS buffer from Section GFP Fluorescence Assays were viewed under the phase-contrast and fluorescence microscopy. Pictures were taken at 100 × magnification, 200 ms.

### Bioinformatics analyses

Genome databases in NCBI (http://www.ncbi.nlm.nih.gov/) and Pfam (http://pfam.sanger.ac.uk/) were used for bioinformatics analyses. Sequence alignments were conducted using web tool Clustal Omega (http://www.ebi.ac.uk/Tools/msa/clustalo/) and the BioEdit Sequence Alignment analytical software, whereas neighbor-joining phylogenetic trees were generated with Composition Vector Tree Version 2 (http://tlife.fudan.edu.cn/cvtree/) and MEGA6.05. A web-based resource for bacterial integrative and conjugative elements (ICEs) termed ICEberg (http://db-mml.sjtu.edu.cn/ICEberg/) (Bi et al., 2012) was used to detect the presence of TAs in pneumococal ICEs. PHAST (PHAge Search Tool) (http://phast.wishartlab.com/) (Zhou et al., 2011) was used to identify TAs in prophage sequences within pneumococcal genomes. In the case of strains, whose sequence types (STs) were not directly available, sequences of seven loci used in the multilocus sequence typing (MLST) scheme (Enright and Spratt, 1998) were retrieved from genomic data and submitted to the online database http://pubmlst.org/spneumoniae/ (28th August 2014, date last accessed) to obtain allele numbers and STs. Position of the STs was then analyzed for all strains within the population snapshot, constructed based on the content of the whole MLST database (28th August 2014, date last accessed) using the eBURST approach (Feil et al., 2004) at http://eburst.mlst.net/ and resulting clonal complexes (CCs) were named based on the predicted founder of the group. The largest observed CC, including ST156 as a proposed primary founder, was additionally divided into lineages named according to the predicted secondary founders.

## Results and discussion

### Verification of the putative TAs from *S. pneumoniae* hungary^19A^-6 in *S. pneumoniae* R6 and in *E. coli* Top10

Between 4 and 10 putative TAs were found in our previous *in silico* analysis of *S. pneumoniae* genomes (Chan et al., 2012). Genes of the putative TAs analyzed here were derived from the Hungary^19A^-6 strain. It is a pathogenic strain, which harbors all 10 putative TA pairs, thus covering most (if not all) of the putative pneumococcal TAs reported to date. Three out of the 10 TA operons, namely the R6 strain *relBE2*, *yefM-yoeB* and *pezAT*, had been previously shown to encode functional TAs in *S. pneumoniae* and also in *E. coli* (Nieto et al., 2006, 2007; Khoo et al., 2007). Thus, these three pairs were not included here, with the exception of the *yoeB* toxin gene that was used as a positive control for the overexpression assays in the *E. coli* host.

**Figure 1:**
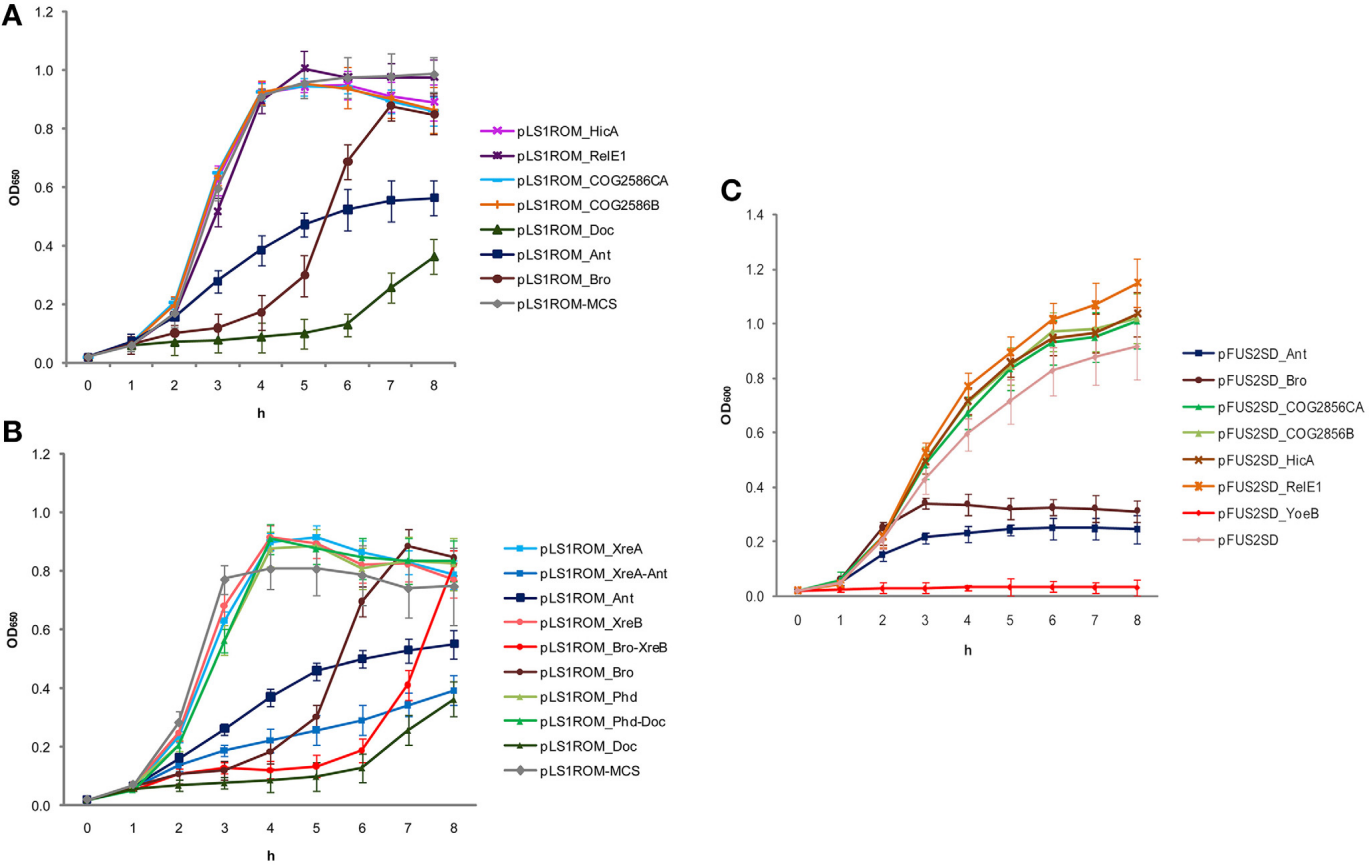
**Overexpression of pneumococcal putative TAs. (A)** Overexpression of pneumococcal putative toxins in homologous host *S. pneumoniae* had shown that HicA, RelE1, COG2856CA, and COG2856C were not harmful to the cells; whereas overexpression of Doc, Ant and Bro had thwarted cell growth but resumed a few hours albeit slowly. **(B)** Since Doc, Ant and Bro were toxic to the cells, they were thus further analyzed to ensure if their putative cognate antitoxins were functional as well in *S. pneumoniae host*. For Doc, co-expression of its cognate antitoxin Phd *in cis* had neutralized the Doc toxicity to the cell, signifying Phd-Doc is a functional TA. On the other hand, overexpression of Ant and Bro repressed the growth of the cells but co-expression of their cognate putative antitoxins XreA and XreB, respectively, were not able to neutralize the toxic effect of the toxins, and instead more toxic to the cells. Note that XreA or XreB alone was inert to the cells. Thus, we have ruled out that XreA-Ant and Bro-XreB are typical TAs. **(C)** Overexpression assays in heterologous host *E. coli*. Similar to the natural host *S. pneumoniae*, overexpression of pneumococcal putative toxins HicA, RelE1, COG2856CA, and COG2856C did not affect cell growth. Unfortunately we were not able to construct doc toxin in pFUS2SD plasmid likely due to its high toxicity. YoeB from strain R6, which is a known functional pneumococcal toxin (Nieto et al., 2007; Chan et al., 2011) was used as a positive control (pFUS2SD_YoeB), whereas pFUS2SD without insert served as a negative control. As with the pneumococcal YoeB toxin, overexpression of pneumococcal Ant and Bro inhibited growth of *E. coli* and no recovery was observed even after 8 h of incubation, which was in contrary to the results in *S. pneumoniae*, in which cell growth was resumed a few hours after toxins overproduction.

The functionality of the seven putative toxins was first analyzed in their natural host *S. pneumoniae* and the ones that retarded cell growth were further analyzed with their cognate antitoxins. In the case of the pneumococcal *doc* gene, the construction of plasmid pLS1ROM-MCS harboring *doc* had a mutation at the SD in which ′AGGAGG′ was changed to ′AGGCGG′. However, this mutation did not hinder Doc protein to exhibit its toxicity to the cell. Overproduction of Doc had strongly retarded the growth of *S. pneumoniae* despite the growth resumed after 6 h (Figure 1A). Nonetheless, its toxicity could be clearly neutralized by co-expression of its cognate antitoxin Phd *in cis* (Figure 1B). This finding demonstrated that the pneumococcal *phd-doc* pair constitutes a *bona fide* functional TA. For Ant and Bro, cell growth was also inhibited when the proteins were overproduced, respectively, albeit less toxic than Doc (Figure 1A). Growth of *S. pneumoniae* cells resumed after a few hours of induction of the toxins (Figure 1A), indicating the toxicity was temporal. Unexpectedly, co-expression of their presumptive cognate antitoxins XreA and XreB did not neutralize the toxicity of Ant and Bro, respectively (Figure 1B). Expression of XreA and XreB alone was not toxic to the cells, but intriguingly, the pairs XreA-Ant and Bro-XreB seemed to be slightly more toxic to the cells, compared to the Ant and Bro toxins alone (Figure 1B). Conversely, overproduction of the rest of the putative pneumococcal toxins HicA, RelE1, COG2856CA, and COG2856B had no effect on the growth of *S. pneumoniae* (Figure 1A).

In the case of the heterologous host *E. coli*, as in their native host *S. pneumoniae*, overexpression of HicA, RelE1, COG2856CA, and COG2856B were inert to the cells (Figure 1C). We were unable to obtain the recombinant plasmid of pFUS2SD with the putative pneumococcal toxin *doc* gene, most likely because of its high toxicity to the host. As it was observed for YoeB, overexpression of the Ant and Bro proteins led to suppression of the growth of *E. coli*; moreover, in contrast to *S. pneumoniae*, no indication that the cultures resumed growth was found even after 8 h of incubation, indicating that both proteins were very toxic to the host (Figure 1C).

We conclude that out of the seven gene pairs that we had investigated here, only Phd-Doc was demonstrated to be a *bona fide* pneumococcal TA. Concerning Ant and Bro, overexpression of each of these proteins inhibited growth of both *S. pneumoniae* and *E. coli*; however, the products of their adjacent genes XreA and XreB were unable to counteract their toxicity, respectively. Therefore, we do not classify them as typical TA pairs. Thus, we have re-evaluated the number of functional *bona fide* TAs for the 48 pneumococcal strains whose genomic sequences are available in the NCBI genome databases (Table 2, see below).

**Table 2:** **Distribution of sequence types, clonal complexes, and functional TAs in *S. pneumonia***.

**Strain (*N* = 48)**	**Sequence type**	**Clonal complex**	**Phd-Doc**	**YefM-YoeB**	**PezAT**	**RelBE2^a^ (type)**	**Total**
INV200	9	15	1	1	1	1 (II)	4
BS397	13	15	1	1	1	1 (II)	4
BS455	2011	15	1	1	1	1 (II)	4
BS457	13	15	1	1	1	1 (II)	4
BS458	13	15	1	1	1	1 (II)	4
SP-BS293	13	15	1	1	1	1 (II)	4
SP14-BS292	13	15	1	1	1	1 (II)	4
CGSP14	15	15	1	1	2	1 (II)	5
AP200	62	62	1	1		1 (II)	3
MLV-016	62	62	1	1		1 (II)	3
SP11-BS70	62	62	1	1		1 (II)	3
G54	63	63	1			1 (II)	2
JJA	66	66	1	1	1	2 (II & V)	5
ATCC 700669	81	81	1		2	2 (II&V)	5
670-6B	90	90	1	1	1	1 (II)	4
GA41317	2705	100		1		1 (II)	2
OXC141	180	180	1		1	1 (II)	3
SP3-BS71	180	180	1		1	1 (II)	3
CDC1087-00	191	191	1	1		1 (II)	3
CDC3059-06	199	199	1		1	2 (I&V)	4
TIGR4	205	205	1	1	1	1 (I)	4
P1031	303	217	1		2	2 (II&VI)	5
CDC0288-04	220	218		1	1	1 (IV)	3
GA41301	242	242	1	1	1	2 (II&V)	5
70585	289	289	1		1	1 (II)	3
INV104	227	306	1	^b^		1 (II)	2
GA47901	304	306	1			1 (II)	2
GA04375	236	320	1			1 (IV)	2
Taiwan19F-14	236	320	1			1 (IV)	2
str. Canada MDR_19A	320	320	1			1 (II)	2
str. Canada MDR_19F	320	320	1			1^c^	2
TCH8431/19A	320	320	1			1 (IV)	2
SP19-BS75	485	395	1	1	1	1 (II)	4
SP23-BS72	37	439		1	1	1 (II)	3
SP6-BS73	460	460	1		1	1 (I)	3
GA17545	9057	1536	1			1 (II)	2
CDC1873-00	376	2090	1	1	1	2 (II&V)	5
GA47368	1339	2090	1	1	1	2 (II&V)	5
CCRI 1974	124	L_124	1			1 (II)	2
CCRI 1974M2	124	L_124	1			1 (II)	2
SP14-BS69	124	L_124	1		1	1 (II)	3
SP18-BS74	146	L_146	1			1 (II)	2
GA17570	156	L_156	1	1	1	1 (III)	4
SP195	156	L_156	1	1	1	1 (III)	4
SP9-BS68	1269	L_162	1	1	1	1 (III)	4
Hungary 19A-6	268	L_176	1	1	1	1 (II)	4
D39	595	singleton		1	1	1 (I)	3
R6	595	singleton		1	1	1 (I)	3

a *The organization types (I–VI) of the RelBE2-TA are indicated within parenthesis*.

b *There is a stop codon within YoeB (only 15 amino acid residues are translated) of strain INV104, and thus YefM-YoeB of this strain was excluded in this table*.

c *Data not available for analyses*.

### The pneumococcal Phd-doc: the fourth functional TA pair in *S. pneumoniae*

**Figure 2:**
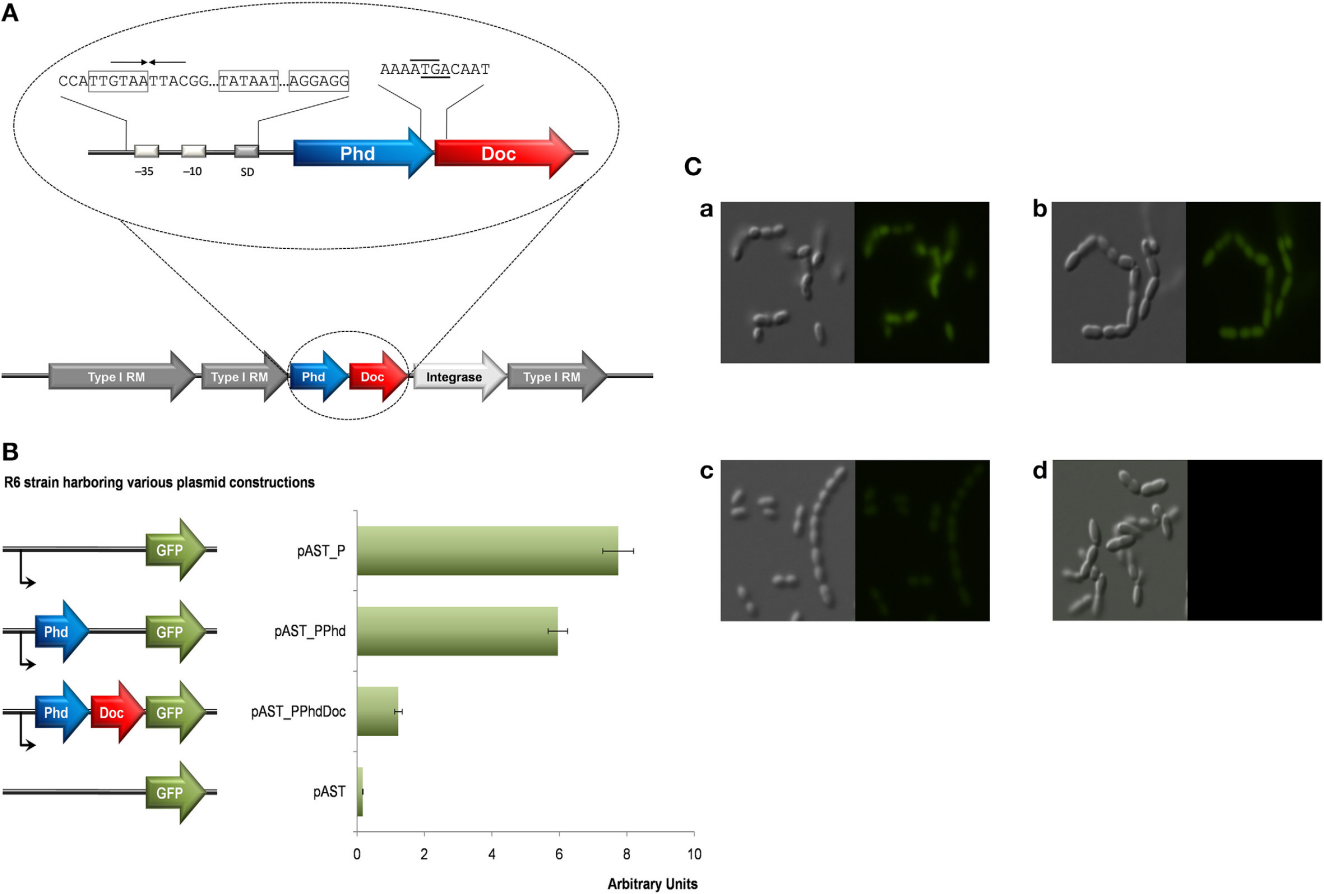
**Gene organization and expression of the pneumococcal *phd-doc* locus. (A)** The pneumococcal *phd-doc* is surrounded by genes that encode Type I restriction-modification (RM) proteins and integrase/recombinase. The pneumococcal *phd* antitoxin gene is located upstream of its cognate toxin gene *doc*, and both genes overlap by 4 nucleotides. A promoter and a Shine-Dalgano (SD) were identified upstream of *phd*, and an inverted repeat (denoted by arrows) was found overlapping the −35 sequences (boxed); the −10 region of the putative promoter and the SD sequences are also boxed. The stop codon of Phd is underlined and the start codon of Doc is indicated in overline. **(B)** Expression and self-regulation of the *phd-doc* operon. Cultures (1 ml) of *S. pneumoniae* R6 cells harboring recombinant plasmids were grown until an OD_650_ ~0.3. Cells were harvested, resuspended in 1 × PBS buffer and their fluorescence was measured. Plasmids used were: pAST_P, harboring only the promoter of the operon; pAST_PPhd, harboring the promoter and the *phd* gene; pAST_PPhdDoc, harboring the entire *phd-doc* operon, and the promoter-less *gfp* plasmid vector, pAST, used as control. *SE* (average of three independent experiments) are indicated. **(C)** Cells examination under phase-contrast and fluorescence microscopy. The GFP level for cells harboring pAST_P (a) and pAST_PPhd (b) did not show much difference; whereas GFP level for pAST_PPhdDoc (c) was visibly lower, which is coincided with the fluorescence measurement. Cells harboring plasmid pAST served as negative control.

As mentioned above, the pneumococcal Doc was shown to be toxic to its natural host, and co-expression of the cognate Phd antitoxin was able to counteract the toxicity of Doc. The pneumococcal *phd-doc* gene pair was present in a single copy in almost all the genomes of pneumococcal strains examined (43 out of 48 strains; Table 2) (Chan et al., 2012). The *phd-doc* genes are structured as an operon, which was consistently surrounded by genes encoding Type I restriction-modification systems; further, a gene that encodes a putative integrase/recombinase was also conserved downstream of *doc* (Figure 2A). However, the genomic context of *phd-doc* was not entirely conserved since, in some strains, transposases were also evident further downstream. The pneumococcal *phd* antitoxin and *doc* toxin genes overlap by four nucleotides and both genes are likely co-transcribed from a single promoter located upstream of *phd* (Figure 2A). An inverted repeat of 8 nt (GTAA·TTAC) was identified, which partly covered the −35 consensus sequences (Figure 2A). This differs from the promoter of *phd-doc* of bacteriophage P1, in which two palindrome sequences (TGTGT·ACACA and CGAGT·ACACG) were found to be located between the −10 consensus sequence and the start codon of Phd, with one of the palindromes overlapping the SD (Magnuson et al., 1996). We postulate that the inverted repeat would function as the operator where the pneumococcal Phd antitoxin binds to repress its own transcription, as in other TAs (Magnuson et al., 1996; Tian et al., 2001; Kedzierska et al., 2007). To test the regulation of transcription of pneumococcal Phd-Doc, we constructed a set of pneumococcal plasmids based on the pAST promoter-probe vector that harbors the *gfp* gene (Ruiz-Cruz et al., 2010). Thus, transcriptional fusions allowed us to measure the *phd-doc* promoter. In addition, and to demonstrate whether Phd antitoxin and the Phd-Doc pair regulate their transcription, either the antitoxin or the TA pair was fused to the promoter-less gene encoding GFP. Measurement of the levels of fluorescence showed that indeed the promoter region resulted in GFP expression of ~8 arbitrary units, which were slightly repressed by synthesis of the antitoxin (~6 arbitrary units) and repressed further to ~1 arbitrary units (down to nearly basal levels ~0.1 arbitrary units) by the joint production of the Phd-Doc pair (Figure 2B). In agreement to these results, observation of these cells under phase-contrast and fluorescence microscopy also demonstrated that the fluorescence was not much different for cells harboring promoter-*gfp* and promoter-*phd-gfp*; whereas the fluorescence of cells harboring promoter-*phd-doc-gfp* was visibly lower (Figure 2C). Employment of the GFP reporter to detect TA activation was previously shown for the *Salmonella sehAB* operon (De La Cruz et al., 2013). In our case, it has turned out to be a simple and reliable way to show auto-repression of the *phd-doc* operon. Further, it could be used for any of the TA to be tested and also to perform *in vivo* studies on conditions that trigger any particular TA. To our understanding, this is the first time that this strategy is performed for a Gram-positive bacterium.

Like other Doc homologs, the pneumococcal Doc has a Fic (filamentation induced by cyclic AMP) domain (Utsumi et al., 1982) at the N-terminal moiety. In general, the conserved Fic motif, HxFx(D/E)GNGRxxR (Engel et al., 2012; Goepfert et al., 2013), possesses adenylylation (or “AMPylation”) activity (Itzen et al., 2011; Woolery et al., 2012). By comparison, the consensus residues of the Fic motifs in all Doc toxins are slightly different, HxFx(D/N)(A/G)NKR (Cruz et al., 2014), as is the pneumococcal Fic motif HVFANANKR. The target of the Doc toxin from bacteriophage P1 (Magnuson and Yarmolinksy, 1998) was revised recently, in which Doc was reported to be a new type of protein kinase [instead of AMPylating like other Fic proteins (Kinch et al., 2009) or obstructing ribosomes (Liu et al., 2008)] that inhibits bacterial translation by phosphorylating the conserved threonine (Thr382) of the translation elongation factor EF-Tu, thereby rendering it unable to bind aminoacylated tRNAs (Castro-Roa et al., 2013; Cruz et al., 2014). Interestingly, it was also demonstrated that the Phd antitoxin can only block the *de novo* phosphorylation of EF-Tu, but cannot revert it, and dephosphorylation of EF-Tu was probably catalyzed by the Doc toxin itself (Castro-Roa et al., 2013). This finding coincided with our observation, in which the inhibition of growth mediated by the pneumococcal Doc toxin was slowly relieved a few hours after overexpression (Figure 1B). It had been shown that ectopic overexpression of Doc in *E. coli* might lead to Lon-dependent activation of RelE to exhibit its mRNA cleavage activity (Garcia-Pino et al., 2008). Since *S. pneumoniae* harbors both RelBE and Phd-Doc TAs, it would be interesting to find out whether both TAs could cross-talk as described by Garcia-Pino et al. (2008).

### Morphology of *E. coli* and *S. pneumoniae* cells after toxin overexpression

**Figure 3:**
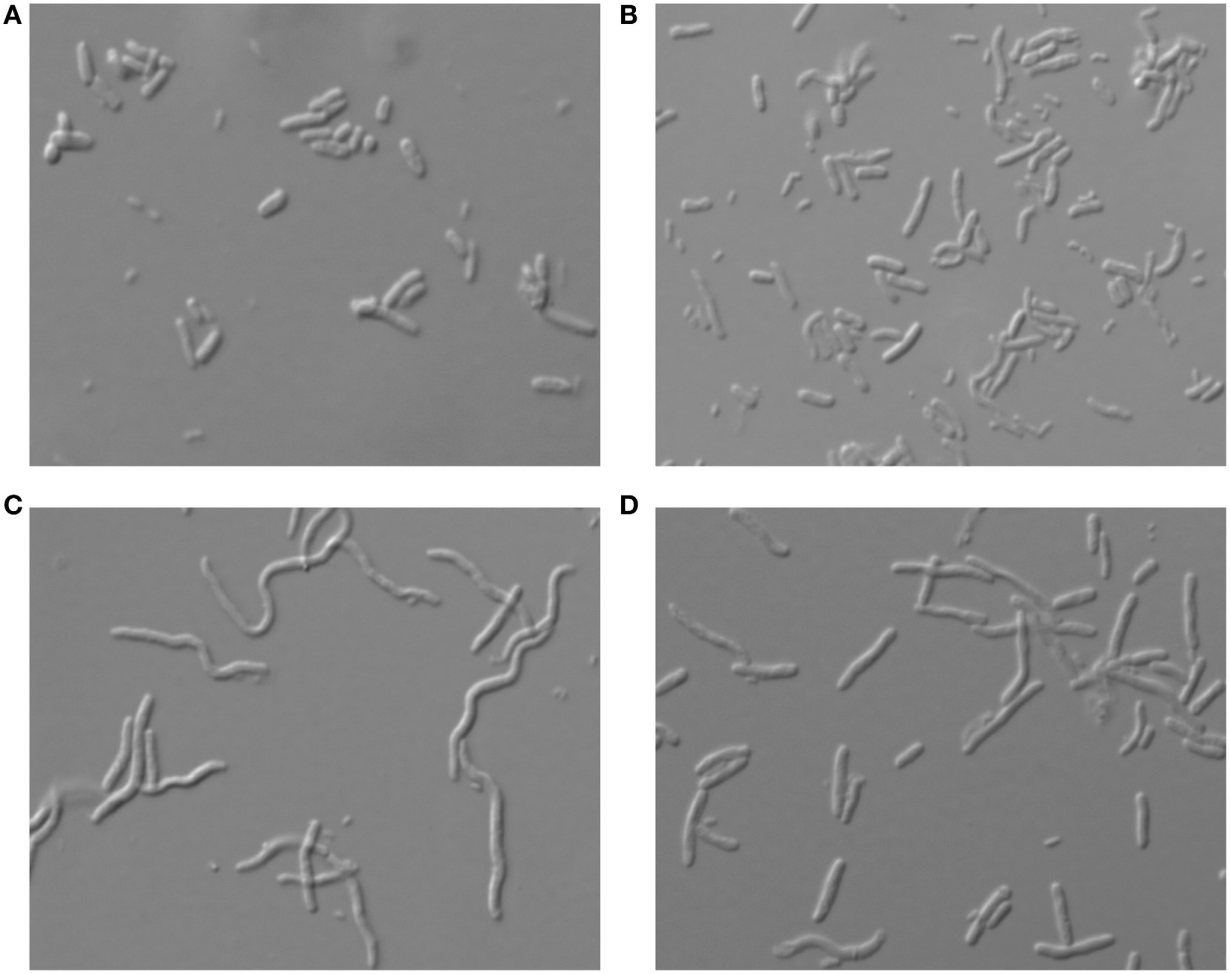
**Morphology of *E. coli* cells under microscope at 100 × magnification after overexpression of the respective pneumococcal toxins**. Morphology of the cells which harbor **(A)** the negative control pFUS2SD, **(B)** the positive control pFUS2SD_YoeB, **(C)** pFUS2SD_Ant and **(D)** pFUS2SD_Bro, were examined after 8 h of overexpression. Filamented cells were observed in cultures overexpressing Ant or Bro, but no prominent differences were found for cells overexpressing YoeB when compared to wild type.

In many cases where overexpressed genes may cause a toxic effect, the bacterial cells change their morphology and may exhibit a phenotype of either filamentation or other gross changes in the cell shape (Scott et al., 2010). To test whether overexpression of the various toxins analyzed here had any influence on the cell morphology of *S. pneumoniae* and *E. coli*, cultures were subjected to toxin overexpression under the above conditions, and cells were observed using phase-contrast microscopy. In the case of *S. pneumoniae* no prominent morphological changes were observed for any cells that overexpressed the different toxins (Supplementary Figure S1). On the other hand, for *E. coli*, filamentation was observed when Ant or Bro were overexpressed, indicating that the cells were not dividing properly and thus cell growth was impaired (Figures 3C,D). The filamentation was not reversible for at least 8 h as inhibition of cell growth by Ant or Bro did not show any sign of resuming even after 8 h (Figure 1C). Overexpression of YoeB toxin, which likely an endoribonuclease as its *E. coli* homolog (Christensen et al., 2004; Zhang and Inouye, 2009), did not result in very obvious differences in morphology when compared to the wild type (Figures 3A,B).

### Other TAs in *S. pneumoniae* and their genetic organization

#### The relBE1 operon

A previous study showed that RelE1 of *S. pneumoniae* had no RNase activity (Christensen and Gerdes, 2003), but the cell growth profile after overexpression of RelE1 was not assessed. Here, we confirmed that expression of RelE1 was not detrimental to its homologous host or to the heterologous host *E. coli* (Figure 1) even though RelE1 shared 52% similarity with RelE2 (in the Hungary^19A^-6 strain) which had been demonstrated to be functional (Nieto et al., 2006). In spite of the high conservation of both *relB1* and *relE1* in all the strains (not shown), two (R65 and R85) out of five crucial residues responsible for RelE toxicity in *Pyrococcus horikoshii* were missing in the pneumococcal RelE1 (Takagi et al., 2005; Nieto et al., 2010; Chan et al., 2012). In addition, truncation of *relE1* in some strains, such as ATCC 700669 and 70585 was also observed. In general, the gene arrangement around the *relBE1* genes of all the strains was similar, i.e., these genes were flanked upstream by genes that encode DNA polymerase III, elongation factor G and 30s ribosomal proteins, whereas downstream genes were found to be encoding a possible amino peptidase, an integral membrane protein and ribosomal small subunit pseudouridine synthase. In some instances, transposases were identified further upstream of *relB1* (strains ATCC 700669 and INV104). Conservation of the *relBE1* gene pair in many of the pneumococcal genomes could point to a function not related to type II TA, but this remains to be investigated.

#### The gene pairs xreA-ant and bro-xreB

These two gene pairs were previously proposed as putative TAs in bioinformatics searches of the *S. pneumoniae* genome (Makarova et al., 1999; Chan et al., 2012). Overexpression of the pneumococcal Ant and Bro proteins inhibited cell growth in both *S. pneumoniae* and *E. coli*, but co-expression of their putative respective cognate genes *xreA* and *xreB* did not counteract their toxicity. However, we still do not rule out the possibility that Ant and Bro are solitary toxins, similar to the MazF-mx of *M. xanthus*, which lacks the co-transcribed antitoxin (Nariya and Inouye, 2008). MrpC, which is expressed from another part of the *M. xanthus* genome, serves as an antitoxin by forming complexes with MazF-mx, and it also positively regulates the MazF-mx expression (Nariya and Inouye, 2008).

**Figure 4:**
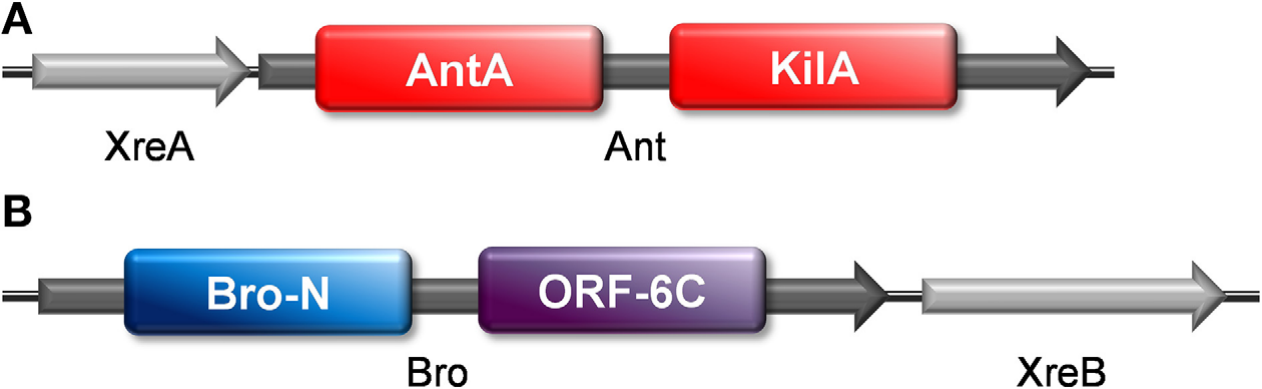
**The domains of pneumococcal Ant and Bro toxins**. Sequence analyses from Pfam showed that **(A)** pneumococcal Ant harbors two domains, with the AntA domain at the N-terminus and KilAC domain at the C-terminus; whereas **(B)** pneumococcal Bro harbors a Bro-N domain at the N-terminal and an ORF6C domain at the C-terminal.

We made use of a web server designed to identify prophages (PHAST; Zhou et al., 2011) to search whether pneumococcal *xreA-ant* and *bro-xreB* gene pairs were located within pneumococcal prophages. We found that they are located within a 94.4 kb intact prophage termed *Streptococcus* phage MM1 of *S. pneumoniae* Hungary^19A^-6 strain (Supplementary material). Analysis with Pfam showed that both Ant and Bro have two domains, respectively: Ant (232 amino acids) has an AntA (AntA/AntB antirepressor) domain at the N-terminal moiety and a phage antirepressor KilAC domain at the C-terminal moiety (Figure 4A); whereas Bro (237 amino acids) has a Bro-N domain at the N-terminus and an ORF6C (C-terminal of bacteriophage bIL2850 ORF6) (Iyer et al., 2002) domain at the C-terminus (Figure 4B). Both KilA and Bro domains are widely prevalent in bacteriophages, bacteria, eukaryotic viruses of the nucleo-cytoplasmic large DNA virus and baculovirus classes (Iyer et al., 2002). A study by Kuan et al. showed that the protein ANT8 (ORF8) of lytic corynephage P1201 isolated from *Corynebacterium glutamicum* harbors a Bro domain at the N-terminus and an antirepressor domain at the C-terminal end (http://research.nchu.edu.tw/upfiles/ADUpload/oc_downmul2272130635.pdf). In this case, the DNA binding ability of ANT8 was contributed by the Bro domain, whereas overexpression of the antirepressor domain inhibited *E. coli* cell growth and also killed the cells as shown by a decrease in colony forming units. Under microscopic examination, the cells that overexpressed the ANT8 protein became filamentous, with irregular multiple nucleoids, indicating inhibition of cell division, and this result is also in agreement with our findings.

### Genomic context of the functional pneumococcal TAs *relBE2* and *pezAT*, and their distribution in *S. pneumoniae* population

**Figure 5:**
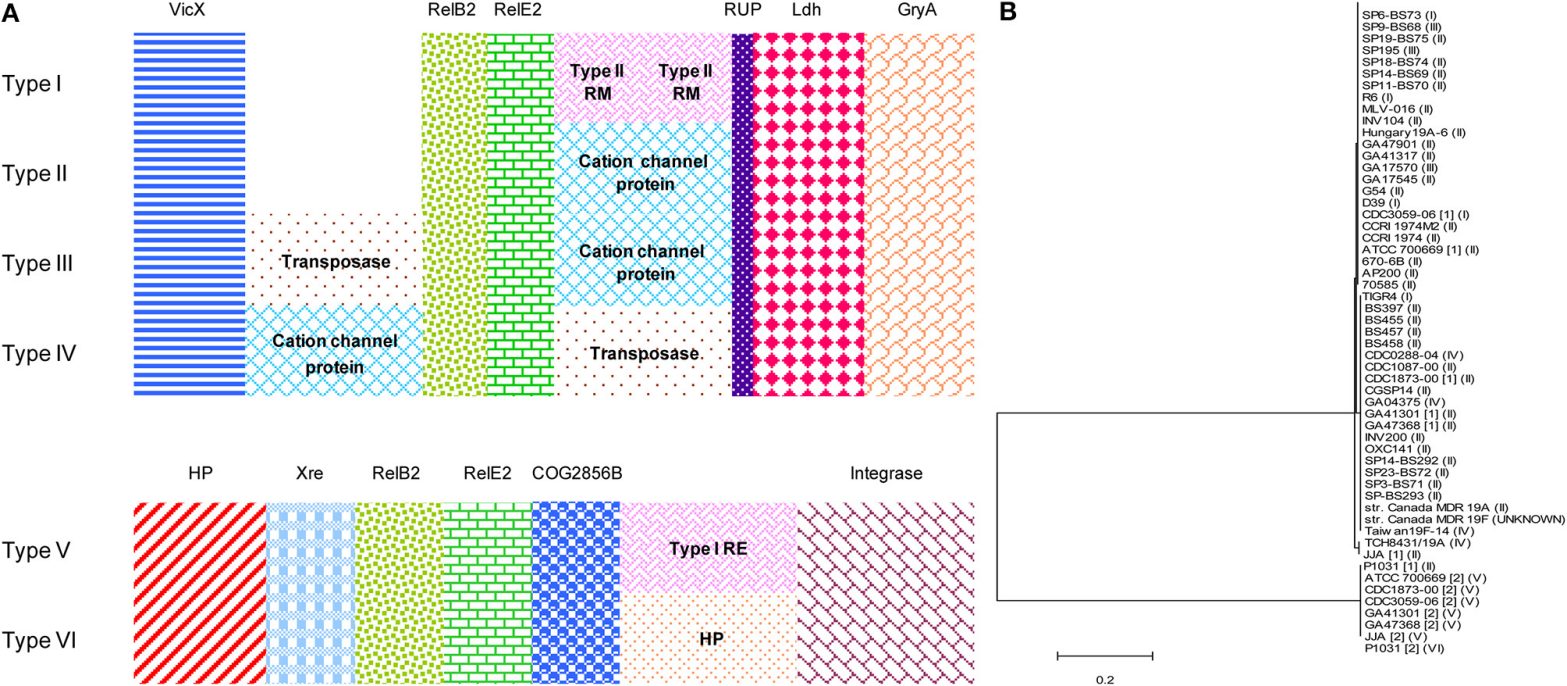
**Gene organization and phylogenetic analyses of pneumococcal RelE2 homologs. (A)** Polymorphisms associated with the gene organization of *relBE2* and their neighboring genes found in 48 pneumococcal strains. Six variations of gene organizations were found (Type I–VI). In general, the *relBE2* operon was flanked upstream by *vicX* (metal-dependent hydrolase), whereas a RUP (repeat unit of pneumococcus), element, *ldh* (lactate dehydrogenease), and *gyrA* (the A subunit of DNA gyrase) are located downstream for Type I–IV. For Type V–VI, the *relBE2* cassette is flanked upstream by *xre*-like protein and COG2856B downstream. Mobile elements are seen in Types III–VI. Other abbreviations used: restriction-modification (RM); restriction-endonuclease (RE); hypothetical protein (HP). **(B)** Phylogenetic analyses of RelE2 homologs with neighbor-joining algorithm, in which the determined start codon of *relE2* was considered (Moreno-Córdoba et al., 2012), had shown that Type V and Type VI gene organizations of RelE2 cluster together, indicating RelE2 from both types are of more similar in sequences. The type of *relBE2* gene organization was indicated. For the strain Canada MDR 19F, the type of gene arrangement is unknown due to data unavailability in the NCBI databases.

The distribution of functional TAs showed a very good correlation with pneumococcal STs and CCs (Table 2). As described in our previous report, RelBE2 is present in all the 48 pneumococcal database strains with seven out of them having two copies. Moreover, six different gene organizations were found for this TA (Chan et al., 2012) that fall into two groups, encompassing the previously defined Types I-IV and Types V-VI (Chan et al., 2012; Figure 5A). The latter are flanked by xre upstream and COG2856B downstream, an arrangement that is not observed in Types I-IV. RelE2 of Types V and VI were also clustered together in the phylogenetic analyses (Figure 5B). In addition, in strains with two copies of RelBE2, one copy belonged to Types I-IV whereas the second copy belonged to either Type V or VI. Integrases and IS elements were evident in Types III-VI. The particular gene arrangement showed also a very good agreement with STs and CCs, with a single exception in the Taiwan^19F^-14 international clone (Table 2). All these observations suggest that the second copy of *relBE2* may likely be horizontally acquired by some strains rather than represent a product of gene duplication.

In our previous bioinformatics search, 28 out of the 48 annotated pneumococcal strains harbored a single copy of *pezAT*; whereas three strains harbored two copies (Chan et al., 2012). The two copies of *pezAT* within the same strain were almost identical (similarities of 96–97%), and the catalytically important residues (Meinhart et al., 2003) were also conserved. The gene organization of *pezAT* among the various *S. pneumoniae* strains is even more varied than *relBE2*. This TA is flanked by a number of genes that contribute to site-specific recombination and even genes that play roles in conjugative transfer. The *pezAT* locus is known to be present on a genomic island known as the pneumococcal pathogenicity island 1 (Meinhart et al., 2003; Nieto et al., 2006) but interestingly, a search using a web-based resource for bacterial ICEs known as ICEberg database led us to discover the presence of a previously unreported *pezAT* operon in a putative pneumococcal ICE designated Tn*5253* (Ayoubi et al., 1991). This was corroborated when a recent analysis of the complete sequence of Tn*5253* from *S. pneumoniae* DP1322 indicated the presence of two copies of *pezAT* (Iannelli et al., 2014) which comprised part of a 1169 bp direct repeat that flanks the Ω *cat*(pC194) element within Tn*5253*. Experimental evidence showed that excision of Ω *cat*(pC194) from Tn*5253* occurred by a recombination event within the *pezAT*-containing repeat segment leaving one copy in Tn*5253* and another copy in the newly-excised plasmid pC194 (Iannelli et al., 2014). Besides that, ectopic integration of Ω *cat*(pC194) to other regions of the *S. pneumoniae* genome was also detected, likely by homologous recombination involving the *pezAT*-containing direct repeat segments (Iannelli et al., 2014). Thus, *pezAT* appeared to play a direct role in the transfer of the Ω *cat*(pC194) element and may also help in the stable maintenance of this element in its hosts. It is therefore not surprising that phylogenetic analyses showed that PezAT found in Tn*5253* formed a separate cluster from PezAT located in the pneumococcal pathogenicity island 1 (Supplementary Figure S2 and Table S1).

### Conclusions

Previous searches for putative type II TAs in *S. pneumoniae* (Chan et al., 2012, 2013) led us to conclude that their number was greatly undervalued. However, our present *in vivo* validation has shown that, indeed, only four of the TAs, namely RelBE2 (Nieto et al., 2006), YefM-YoeB (Nieto et al., 2007; Chan et al., 2011), PezAT (Khoo et al., 2007; Mutschler et al., 2011) and now, Phd-Doc can be considered as *bona fide* TAs in the “classical” sense that the harmful effects of the toxins must be counteracted by their cognate antitoxins and that their two genes should be organized as an operon (Gerdes, 2013). Bioinformatics search approaches have helped us to identify various putative TA homologs; however, the actual number of *bona fide* TAs might be overestimated as in our case e.g., pneumococcal *relBE2* is functional TA but pneumococcal *relBE1* is apparently not. In the case of *M. tuberculosis*, of the 88 putative TAs identified, only 30 are functional (Ramage et al., 2009). Other approaches like shotgun cloning as presented by Sberro et al. (2013) could be another alternative and more thorough approach to identify TAs. However, there might be more TAs than these four if we consider Ant and Bro as possible solo toxins whose respective antitoxins might be located elsewhere in the pneumococcal chromosome. The finding that these two novel proteins might be associated with lysogenic bacteriophages is, indeed, appealing and merits an in-depth study of their features.

## Author contributions

Manuel Espinosa, Chew Chieng Yeo, Ewa Sadowy and Wai Ting Chan designed the study. Wai Ting Chan performed laboratory work and data analysis. Manuel Espinosa, Chew Chieng Yeo, Ewa Sadowy and Wai Ting Chan wrote the manuscript. All authors read and approved the final manuscript.

### Conflict of interest statement

The reviewer Ramon Diaz Orejas declares that, despite being affiliated at the same institution as the authors Wai Ting Chan and Manuel Espinosa, the review process was handled objectively and no conflict of interest exists. The authors declare that the research was conducted in the absence of any commercial or financial relationships that could be construed as a potential conflict of interest.

## Abbreviations

TAs: toxins-antitoxins
SD: Shine-Dalgarno
*ant*: *antirepressor*
*bro*: *baculovirus repeated orfs*
GFP: Green Fluorescent Protein
OD: optical density
STs: sequence types
ICEs: integrative and conjugative elements
MLST: multilocus sequence typing
CCs: clonal complexes
Fic: filamentation induced by cyclic AMP.

## References

[B1] Ayoubi P. Kilic A. O. Vijayakumar M. N. (1991). Tn*5253*, the pneumococcal omega (*cat tet*) BM6001 element, is a composite structure of two conjugative transposons, Tn*5251* and Tn*5252*. J. Bacteriol. 173, 1617–1622. 1847905 10.1128/jb.173.5.1617-1622.1991 PMC207310

[B2] Baquero F. (2009). Environmental stress and evolvability in microbial systems. Clin. Microbiol. Infect. 15, 5–10. 10.1111/j.1469-0691.2008.02677.x 19220344

[B3] Bi D. Xu Z. Harrison E. M. Tai C. Wei Y. He X.. (2012). ICEberg: a web-based resource for integrative and conjugative elements found in Bacteria. Nucleic Acids Res. 40, D621–D626. 10.1093/nar/gkr846 22009673 PMC3244999

[B4] Castro-Roa D. Garcia-Pino A. De Gieter S. Van Nuland N. A. J. Loris R. Zenkin N. (2013). The Fic protein Doc uses an inverted substrate to phosphorylate and inactivate EF-Tu. Nat. Chem. Biol. 9, 811–817. 10.1038/nchembio.1364 24141193 PMC3836179

[B5] Chan W. T. Moreno-Córdoba I. Yeo C. C. Espinosa M. (2012). Toxin-antitoxin genes of the gram-positive pathogen *Streptococcus pneumoniae*: so few and yet so many. Microbiol. Mol. Biol. Rev. 76, 773–791. 10.1128/MMBR.00030-12 23204366 PMC3510519

[B6] Chan W. T. Moreno-Córdoba I. Yeo C. C. Espinosa M. (2013). Toxin-antitoxin loci in *Streptococcus pneumoniae*, in Prokaryotic toxin-antitoxins, ed Gerdes K. (Berlin: Springer-Verlag Berlin Heidelberg), 315–339 10.1007/978-3-642-33253-1_18

[B7] Chan W. T. Nieto C. Harikrishna J. A. Khoo S. K. Yasmin Othman R. Espinosa M.. (2011). Genetic regulation of the *yefM-yoeB_Spn_* toxin-antitoxin locus of *Streptococcus pneumoniae*. J. Bacteriol. 193, 4612–4625. 10.1128/JB.05187-11 21764929 PMC3165651

[B8] Christensen K. S. Maenhauf-Michel G. Mine N. Gothesman S. Gerdes K. Van Melderen L. (2004). Overproduction of the Lon protease triggers inhibition of translation in *Escherichia coli*: involvement of the *yefM-yoeB* toxin-antitoxin system. Mol. Microbiol. 51, 1705–1717. 10.1046/j.1365-2958.2003.03941.x 15009896

[B9] Christensen S. K. Gerdes K. (2003). RelE toxins from bacteria and archaea cleave mRNAs on translating ribosomes, which are rescued by tmRNA. Mol. Microbiol. 48, 1389–1400. 10.1046/j.1365-2958.2003.03512.x 12787364

[B10] Christensen S. K. Mikkelsen M. Pedersen K. Gerdes K. (2001). RelE, a global inhibitor of translation, is activated during nutritional stress. Proc. Natl. Acad. Sci. U.S.A. 98, 14328–14333. 10.1073/pnas.251327898 11717402 PMC64681

[B11] Claverys J. P. Prudhomme M. Martin B. (2006). Induction of competence regulons as general stress responses in Gram-positive bacteria. Annu. Rev. Microbiol. 60, 451–475. 10.1146/annurev.micro.60.080805.142139 16771651

[B12] Cruz J. W. Rothenbacher F. P. Maehigashi T. Lane W. S. Dunham C. M. Woychik N. A. (2014). Doc toxin is a kinase that inactivates elongation factor Tu. J. Biol. Chem. 289, 7788–7798. 10.1074/jbc.M113.544429 24448800 PMC3953291

[B13] De La Cruz M. A. Zhao W. Farenc C. Gimenez G. Raoult D. Cambillau C.. (2013). A toxin-antitoxin module of *Salmonella* promotes virulence in mice. PLoS Pathog. 9:e1003827. 10.1371/journal.ppat.1003827 24385907 PMC3868539

[B14] Dy R. L. Przybilski R. Semeijn K. Salmond G. P. C. Fineran P. C. (2014). A widespread bacteriophage abortive infection system functions through a Type IV toxin–antitoxin mechanism. Nucleic Acids Res. 42, 4590–4605. 10.1093/nar/gkt1419 24465005 PMC3985639

[B15] Engel P. Goepfert A. Stanger F. V. Harms A. Schmidt A. Schirmer T.. (2012). Adenylylation control by intra- or intermolecular active-site obstruction in Fic proteins. Nature 482, 107–110. 10.1038/nature10729 22266942

[B16] Engelberg-Kulka H. Glaser G. (1999). Addiction modules and programmed cell death and antideath in bacterial cultures. Annu. Rev. Microbiol. 53, 43–70. 10.1146/annurev.micro.53.1.43 10547685

[B17] Enright M. C. Spratt B. G. (1998). A multilocus sequence typing scheme for *Streptococcus pneumoniae*: identification of clones associated with serious invasive disease. Microbiology 144, 3049–3060. 10.1099/00221287-144-11-3049 9846740

[B18] Espinosa M. (2013). Plasmids as models to study macromolecular interactions: the pMV158 paradigm. Res. Microbiol. 164, 199–204. 10.1016/j.resmic.2013.01.006 23385144

[B19] Feil E. J. Li B. C. Aanensen D. M. Hanage W. P. Spratt B. G. (2004). eBURST: inferring patterns of evolutionary descent among clusters of related bacterial genotypes from multilocus sequence typing data. J. Bacteriol. 186, 1518–1530. 10.1128/JB.186.5.1518-1530.2004 14973027 PMC344416

[B20] Fineran P. C. Blower T. R. Foulds I. J. Humphreys D. P. Lilley K. S. Salmond G. P. (2009). The phage abortive infection system, ToxIN, functions as a protein-RNA toxin-antitoxin pair. Proc. Natl. Acad. Sci. U.S.A. 106, 894–899. 10.1073/pnas.0808832106 19124776 PMC2630095

[B21] Garcia-Pino A. Christensen-Dalsgaard M. Wyns L. Yarmolinsky M. B. Magnuson R. D. Gerdes K.. (2008). Doc of prophage P1 is inhibited by its antitoxin partner Phd through fold complementation. J. Biol. Chem. 283, 30821–30827. 10.1074/jbc.M805654200 18757857 PMC2576525

[B22] Gerdes K. (ed.). (2013). Prokaryotic Toxin-Antitoxins. Berlin-Heidelberg: Springer 10.1007/978-3-642-33253-1

[B23] Gerdes K. Maisonneuve E. (2012). Bacterial persistence and toxin-antitoxin loci. Annu. Rev. Microbiol. 66, 103–123. 10.1146/annurev-micro-092611-150159 22994490

[B24] Goeders N. Van Melderen L. (2014). Toxin-antitoxin systems as multilevel interaction systems. Toxins 6, 304–324. 10.3390/toxins6010304 24434905 PMC3920263

[B25] Goepfert A. Stanger F. V. Dehio C. Schirmer T. (2013). Conserved inhibitory mechanism and competent ATP binding mode for adenylyltransferases with Fic fold. PLoS ONE 8:e64901. 10.1371/journal.pone.0064901 23738009 PMC3667792

[B26] Hall T. A. (1999). BioEdit: a user-friendly biological sequence alignment editor and analysis program for Windows 95/98/NT. Nucleic Acids Symp. Ser. 41, 95–98.

[B27] Harrison J. J. Wade W. D. Akierman S. Vacchi-Suzzi C. Stremick C. A. Turner R. J.. (2009). The chromosomal toxin *yafQ* is a determinant of multidrug tolerance for *Escherichia coli* growing in a biofilm. Antimicrob. Agents Chemother. 53, 2253–2258. 10.1128/AAC.00043-09 19307375 PMC2687228

[B28] Hoskins J. Alborn W. E. Jr. Arnold J. Blaszczak L. C. Burgett S. Dehoff B. S.. (2001). Genome of the bacterium *Streptococcus pneumoniae* strain R6. J. Bacteriol. 183, 5709–5717. 10.1128/JB.183.19.5709-5717.2001 11544234 PMC95463

[B29] Iannelli F. Santoro F. Oggioni M. R. Pozzi G. (2014). Nucleotide sequence analysis of integrative conjugative element Tn*5253* of *Streptococcus pneumoniae*. Antimicrob. Agents Chemother. 58, 1235–1239. 10.1128/AAC.01764-13 24295984 PMC3910829

[B30] Itzen A. Blankenfeldt W. Goody R. S. (2011). Adenylylation: renaissance of a forgotten post-translational modification. Trends Biochem. Sci. 36, 221–228. 10.1016/j.tibs.2010.12.004 21256032

[B31] Iyer L. M. Koonin E. V. Aravind L. (2002). Extensive domain shuffling in transcription regulators of DNA viruses and implications for the origin of fungal APSES transcription factors. Genome Biol. 3, RESEARCH0012. 10.1186/gb-2002-3-3-research0012 11897024 PMC88810

[B32] Jørgensen M. G. Pandey D. P. Jaskolska M. Gerdes K. (2009). HicA of *Escherichia coli* defines a novel family of translation-independent mRNA interferases in bacteria and archaea. J. Bacteriol. 191, 1191–1199. 10.1128/JB.01013-08 19060138 PMC2631989

[B33] Kedzierska B. Lian L. Y. Hayes F. (2007). Toxin–antitoxin regulation: bimodal interaction of YefM–YoeB with paired DNA palindromes exerts transcriptional autorepression. Nucleic Acids Res. 35, 325–339. 10.1093/nar/gkl1028 17170003 PMC1802561

[B34] Khoo S. K. Loll B. Chan W. T. Shoeman R. L. Ngoo L. Yeo C. C.. (2007). Molecular and structural characterization of the PezAT chromosomal toxin-antitoxin system of the human pathogen *Streptococcus pneumoniae*. J. Biol. Chem. 282, 19606–19618. 10.1074/jbc.M701703200 17488720

[B35] Kinch L. N. Yarbrough M. L. Orth K. Grishin N. V. (2009). Fido, a novel AMPylation domain common to *fic*, *doc*, and AvrB. PLoS ONE 4:e5818. 10.1371/journal.pone.0005818 19503829 PMC2686095

[B36] Lacks S. (1968). Genetic regulation of maltosaccharide utilization in Pneumococcus. Genetics 60, 685–706. 4389668 10.1093/genetics/60.4.685 PMC1212124

[B37] Lacks S. A. López P. Greenberg B. Espinosa M. (1986). Identification and analysis of genes for tetracycline resistance and replication functions in the broad-host-range plasmid pLS1. J. Mol. Biol. 192, 753–765. 10.1016/0022-2836(86)90026-4 2438417

[B38] Lemonnier M. Ziegelin G. Reick T. Muñoz Gómez A. Díaz-Orejas R. Lanka E. (2003). P1 Ban protein is a hexameric DNA helicase that interacts with and substitutes for *Escherichia coli* DnaB. Nucleic Acid Res. 31, 3918–3928. 10.1093/nar/gkg463 12853607 PMC165978

[B39] Leplae R. Geeraerts D. Hallez R. Guglielmini J. Drèze P. Van Melderen L. (2011). Diversity of bacterial type II toxin–antitoxin systems: a comprehensive search and functional analysis of novel families. Nucleic Acids Res. 39, 5513–5525. 10.1093/nar/gkr131 21422074 PMC3141249

[B40] Liu M. Zhang Y. Inouye M. Woychik N. A. (2008). Bacterial addiction module toxin Doc inhibits translation elongation through its association with the 30S ribosomal subunit. Proc. Natl. Acad. Sci. U.S.A. 105, 5885–5890. 10.1073/pnas.0711949105 18398006 PMC2311363

[B41] Magnuson R. Lehnherr H. Mukhopadhyay G. Yarmolinsky M. B. (1996). Autoregulation of the plasmid addiction operon of bacteriophage P1. J. Biol. Chem. 271, 18705–18710. 10.1074/jbc.271.31.18705 8702525

[B42] Magnuson R. Yarmolinksy M. B. (1998). Corepression of the P1 addiction operon by Phd and Doc. J. Bacteriol. 180, 6342–6351. 9829946 10.1128/jb.180.23.6342-6351.1998 PMC107722

[B43] Makarova K. S. Aravind L. Galperin M. Y. Grishin N. V. Tatusov R. L. Wolf Y. I.. (1999). Comparative genomics of the Archaea (Euryarchaeota): evolution of conserved protein families, the stable core, and the variable shell. Genome Res. 9, 608–628. 10413400

[B44] Makarova K. Wolf Y. I. Koonin E. V. (2009). Comprehensive comparative-genomic analysis of type 2 toxin-antitoxin systems and related mobile stress response systems in prokaryotes. Biol. Direct 4:19. 10.1186/1745-6150-4-19 19493340 PMC2701414

[B45] Maniatis T. Fritsch E. F. Sambrook J. (1982). Molecular Cloning: A Laboratory Manual. New York, NY: Cold Spring Harbor Laboratory Press.

[B46] McGee L. McDougal L. Zhou J. Spratt B. G. Tenover F. C. George R.. (2001). Nomenclature of major antimicrobial-resistant clones of *Streptococcus pneumoniae* defined by the pneumococcal molecular epidemiology network. J. Clin. Microbiol. 39, 2565–2571. 10.1128/JCM.39.7.2565-2571.2001 11427569 PMC88185

[B47] Meinhart A. Alonso J. C. Strater N. Saenger W. (2003). Crystal structure of the plasmid maintenance system *epsilon/zeta*: functional mechanism of toxin zeta and inactivation by *epsilon2zeta2* complex formation. Proc. Natl. Acad. Sci. U.S.A. 100, 1661–1666. 10.1073/pnas.0434325100 12571357 PMC149889

[B48] Moreno-Córdoba I. Diago-Navarro E. Barendregt A. Heck A. J. R. Alfonso C. Díaz-Orejas R.. (2012). The toxin-antitoxin proteins RelBE2*Spn* of *Streptococcus pneumoniae*: characterization and association to their DNA target. Proteins 80, 1834–1846. 10.1002/prot.24081 22488579

[B49] Mutschler H. Gebhardt M. Shoeman R. L. Meinhart A. (2011). A novel mechanism of programmed cell death in bacteria by toxin-antitoxin systems corrupts peptidoglycan synthesis. PLoS Biol. 9:e1001033. 10.1371/journal.pbio.1001033 21445328 PMC3062530

[B50] Nariya H. Inouye M. (2008). MazF, an mRNA interferase, mediates programmed cell death during multicellular *Myxococcus* development. Cell 132, 55–66. 10.1016/j.cell.2007.11.044 18191220

[B51] Nieto C. Cherny I. Khoo S. K. García De Lacoba M. Chan W. T. Yeo C. C.. (2007). The *yefM-yoeB* toxin-antitoxin systems of *Escherichia coli* and *Streptococcus pneumoniae*: functional and structural correlation. J. Bacteriol. 189, 1266–1278. 10.1128/JB.01130-06 17071753 PMC1797350

[B52] Nieto C. Pellicer T. Balsa D. Christensen S. K. Gerdes K. Espinosa M. (2006). The chromosomal *relBE*2 toxin-antitoxin locus of *Streptococcus pneumoniae*: characterization and use of a bioluminescence resonance energy transfer assay to detect toxin-antitoxin interaction. Mol. Microbiol. 59, 1280–1296. 10.1111/j.1365-2958.2006.05027.x 16430700

[B53] Nieto C. Sadowy E. De La Campa A. G. Hryniewicz W. Espinosa M. (2010). The *relBE2Spn* toxin-antitoxin system of *Streptococcus pneumoniae*: role in antibiotic tolerance and functional conservation in clinical isolates. PLoS ONE 5:e11289. 10.1371/journal.pone.0011289 20585658 PMC2890582

[B54] Norton J. P. Mulvey M. A. (2012). Toxin-antitoxin systems are important for niche-specific colonization and stress resistance of uropathogenic *Escherichia coli*. PLoS Pathog. 8:e1002954. 10.1371/journal.ppat.1002954 23055930 PMC3464220

[B55] Pandey D. P. Gerdes K. (2005). Toxin-antitoxin loci are highly abundant in free-living but lost from host-associated prokaryotes. Nucleic Acids Res. 33, 966–976. 10.1093/nar/gki201 15718296 PMC549392

[B56] Ramage H. R. Connolly L. E. Cox J. S. (2009). Comprehensive functional analysis of *Mycobacterium tuberculosis* toxin-antitoxin systems: implications for pathogenesis, stress responses, and evolution. PLoS Genet. 5:e1000767. 10.1371/journal.pgen.1000767 20011113 PMC2781298

[B57] Ren D. Walker A. Daines D. A. (2012). Toxin-antitoxin loci *vapBC-1* and *vapXD* contribute to survival and virulence in nontypeable *Haemophilus influenzae*. BMC Microbiol. 12:263. 10.1186/1471-2180-12-263 23157645 PMC3560280

[B58] Rowe-Magnus D. A. Guerout A. M. Biskri L. Bouige P. Mazel D. (2003). Comparative analysis of superintegrons: engineering extensive genetic diversity in the Vibrionaceae. Genome Res. 13, 428–442. 10.1101/gr.617103 12618374 PMC430272

[B59] Ruiz-Cruz S. Solano-Collado V. Espinosa M. Bravo A. (2010). Novel plasmid-based genetic tools for the study of promoters and terminators in *Streptococcus pneumoniae* and *Enterococcus faecalis*. J. Microbiol. Methods 83, 156–163. 10.1016/j.mimet.2010.08.004 20801171

[B60] Ruiz-Masó J. A. López-Aguilar C. Nieto C. Sanz M. Burón P. Espinosa M.. (2012). Construction of a plasmid vector based on the pMV158 replicon for cloning and inducible gene expression in *Streptococcus pneumoniae*. Plasmid 67, 53–59. 10.1016/j.plasmid.2011.09.001 21946126

[B61] Sambrook J. Russel D. W. (2001). Molecular Cloning: A Laboratory Manual. New York, NY: Cold Spring Harbor Laboratory Press.

[B62] Sberro H. Leavitt A. Kiro R. Koh E. Peleg Y. Qimron U.. (2013). Discovery of functional toxin/antitoxin systems in bacteria by shotgun cloning. Mol. Cell 50, 136–148. 10.1016/j.molcel.2013.02.002 23478446 PMC3644417

[B63] Schifano J. M. Woychik N. A. (2014). 23S rRNA as an a-Maz-ing new bacterial toxin target. RNA Biol. 11, 101–105. 10.4161/rna.27949 24525465 PMC3973728

[B64] Scott M. Gunderson C. W. Mateescu E. M. Zhang Z. Hwa T. (2010). Interdependence of cell growth and gene expression: origins and consequences. Science 330, 1099–1102. 10.1126/science.1192588 21097934

[B65] Soo V. W. Wood T. K. (2013). Antitoxin MqsA represses curli formation through the master biofilm regulator CsgD. Sci. Rep. 3, 3186. 10.1038/srep03186 24212724 PMC4894380

[B66] Szekeres S. Dauti M. Wilde C. Mazel D. Rowe-Magnus D. A. (2007). Chromosomal toxin-antitoxin loci can diminish large-scale genome reductions in the absence of selection. Mol. Microbiol. 63, 1588–1605. 10.1111/j.1365-2958.2007.05613.x 17367382

[B67] Takagi H. Kakuta Y. Okada T. Yao M. Tanaka I. Kimura M. (2005). Crystal structure of archaeal toxin-antitoxin RelE-RelB complex with implications for toxin activity and antitoxin effects. Nat. Struct. Mol. Biol. 12, 327–331. 10.1038/nsmb911 15768033

[B68] Tian Q.-B. Ohnishi M. Murata T. Nakayama K. Terawaki Y. Hayashi T. (2001). Specific protein-DNA and protein-protein interaction in the *hig* gene system, a plasmid-borne proteic killer gene system of plasmid Rts1. Plasmid 45, 63–74. 10.1006/plas.2000.1506 11322821

[B69] Utsumi R. Nakamoto Y. Kawamukai M. Himeno M. Komano T. (1982). Involvement of cyclic AMP and its receptor protein in filamentation of an *Escherichia coli fic* mutant. J. of Bacteriol. 151, 807–812. 6284712 10.1128/jb.151.2.807-812.1982 PMC220329

[B70] Van Melderen L. (2002). Molecular interactions of the CcdB poison with its bacterial target, the DNA gyrase. Int. J. Med. Microbiol. 291, 537–544. 10.1078/1438-4221-00164 11890555

[B71] Woolery A. R. Luong P. Broberg C. A. Orth K. (2012). AMPylation: something old is new again. Front Microbiol. 1:113. 10.3389/fmicb.2010.00113 21607083 PMC3095399

[B72] Yamaguchi Y. Inouye M. (2009). mRNA interferases, sequence-specific endoribonucleases from the toxin-antitoxin systems. Prog. Mol. Biol. Transl. Sci. 85, 467–500. 10.1016/S0079-6603(08)00812-X 19215780

[B73] Zhang Y. Inouye M. (2009). The inhibitory mechanism of protein synthesis by YoeB, an *Escherichia coli* toxin. J. Biol. Chem. 284, 6627–6638. 10.1074/jbc.M808779200 19124462 PMC2652283

[B74] Zhou Y. Liang Y. Lynch K. H. Dennis J. J. Wishart D. S. (2011). PHAST: a fast phage search tool. Nucleic Acids Res. 39, W347–W352. 10.1093/nar/gkr485 21672955 PMC3125810

