# Supplementary material for: Functional validation of putative toxin-antitoxin genes from the Gram-positive pathogen Streptococcus pneumoniae: phd-doc is the fourth bona-fide operon
Source: Front Microbiol. 2014 Dec 5;5:677. doi: 10.3389/fmicb.2014.00677 (PMC4257102; doi:10.3389/fmicb.2014.00677)
Supplement: Supplementary file 1 [file DataSheet1.DOC]

***Supplementary Material***

# **Functional validation of putative toxin-antitoxin genes from the Gram-positive pathogen *Streptococcus pneumoniae*: *phd-doc* is the fourth *bona-fide* operon**

**Wai Ting Chan^1^*, Chew Chieng Yeo^2^, Ewa Sadowy^3^, and Manuel Espinosa^1^**

^1^Centro de Investigaciones Biológicas, Consejo Superior de Investigaciones Científicas, Madrid, Spain

^2^Faculty of Medicine and Health Sciences, Universiti Sultan Zainal Abidin, Kuala Terengganu, Terengganu, Malaysia

^3^National Medicines Institute, Warsaw, Poland

***Correspondence:** Wai Ting Chan, Centro de Investigaciones Biológicas, Consejo Superior de Investigaciones Científicas, Ramiro de Maeztu 9, 28040 Madrid, Spain

chanyting@hotmail.com

The pneumococcal *xreA-ant* and *bro-xreB* gene pairs are located within a 94.4 kb intact prophage termed *Streptococcus* phage MM1 of *S. pneumoniae* Hungary^19A^-6 strain. Due to their genetic organization, we predicted that they may constitute novel pneumococcal TAs (Chan et al., 2012). We have found two domains in the pneumococcal Ant and Bro proteins, respectively. The pneumococcal Ant (232 amino acids) showed an N-terminally located AntA (AntA/AntB antirepressor) domain and a C-terminal antirepressor KilAC domain. The pneumococcal Bro (237 amino acids) showed a Bro-N domain at the N-terminus and an ORF6C (C-terminal of bacteriophage bIL2850 ORF6) (Iyer et al., 2002) domain at the C-terminus. We also discovered similarities between both domains in terms of phyletic patterns, intragenomic distribution and domain architecture. These two domains seemed to be DNA-binding domains: mutations to the KilA-N domain of N1R protein affected its localization to viral DNA-containing cytoplasmic virus maturation complexes (Brick et al., 1998); *Bombyx mori nucleopolyhedrovirus* (BmNPV) Bro proteins can bind DNA and have higher affinity to single-stranded DNA than double-stranded DNA (Zemskov et al., 2000). The KilA and Bro domains are always located at one end of the protein and fused to another distinct domain at the other terminus (Iyer et al., 2002), as it was in the case of the Ant and Bro proteins of *S. pneumoniae*.

For the pneumococcal XreA-Ant, the sequence of the AntA and KilAC domains within the Ant protein shared some degree of similarity between each other (39% similarity). The *Escherichia coli* bacteriophage P1 harbours two *ant* genes (*ant1* and *ant2*, with *ant2* within the 3´ reading frame of *ant1*) and one *kilA* gene. The Ant protein of bacteriophage P1 is an antirepressor that inactivates the C1 repressor protein which represses the lytic genes (Yarmolinsky and Sternberg, 1988;Riedel et al., 1993;Biere et al., 2014). The pneumococcal Ant bears resemblance to both Ant2 and KilA proteins (~48% similarity, in both cases) of phage P1, and thus it is tempting to speculate that the pneumococcal Ant could similarly serve as a phage antirepressor protein. However, the organisation of the neighbouring genes of the pneumococcal *ant* is different from those of the phage P1 *ant1/ant2* and *kilA*. The putative XreA protein, which has a helix-turn-helix motif (and which is not found in neighbouring regions of the P1 Ant1/Ant2), was encoded one nucleotide upstream of the pneumococcal *ant*, and is thus likely co-transcribed together with *ant*. Since XreA does not appear to be the antitoxin for the pneumococcal Ant in this study, its function remains enigmatic, although we could speculate that XreA, having a helix-turn-helix motif, might regulate expression of Ant.

As for the pneumococcal *bro-xreB*, the *bro* gene is located 12 nucleotides upstream of *xreB*. XreB is an unknown protein with no motif found using Pfam. There are five *bro* genes found in BmNPV (Gomi et al., 1999;Kang et al., 1999) and their promoters are well conserved. Although the TATA box consensus sequence was not found in the promoters of BmNPV *bro* genes, a conserved consensus baculovirus early-gene promoter motif C(T)AGT was found 50-70 nucleotides upstream of the Bro protein start codon (Kang et al., 1999). Strikingly, studies had shown that the CAGT motif is sufficient for proper transcription in a TATA-independent manner (Pullen and Friesen, 1995). Interestingly, the CAGT motif was also spotted 75 nucleotides upstream of the pneumococcal Bro start codon. However, a putative bacterial ‒10 promoter sequences AATAAT and a putative ‒35 sequences TTGTTA with a spacer of 18-nucleotides were also identified, albeit 229 nucleotides upstream of the Bro start codon. Activation of the BmNPV *bro* promoter was shown to be dependent on viral factor(s) and *bro* was expressed in the early stage of infection (Kang et al., 1999). Besides binding to DNA, BmNPV Bro proteins can also avidly associate with chromatin, and were shown to interact with core histones (Zemskov et al., 2000). Hence, Bro proteins were suggested to be delayed-early protein, functioning to block cellular replication or transcription and thereby helping to switch the host machinery to viral DNA or RNA synthesis by binding to the host chromosomal DNA (Zemskov et al., 2000). For the pneumococcal Bro protein, it also has a fused ORF6C domain at the C-terminal, in addition to the Bro-N domain. The function of ORF6C is currently unknown.

**References**

Biere, A.L., Citron, M., and Schuster, H. (2014). Transcriptional control via translational repression by c4 antisense RNA of bacteriophages P1 and P7. *Genes Dev.* 6**,** 2409-2416.

Brick, D.J., Burke, R.D., Schiff, L., and Upton, C. (1998). Shope fibroma virus RING finger protein N1R binds DNA and inhibits apoptosis. *Virology* 249**,** 42-51.

Chan, W.T., Moreno-Córdoba, I., Yeo, C.C., and Espinosa, M. (2012). Toxin-antitoxin genes of the gram-positive pathogen *Streptococcus pneumoniae*: so few and yet so many. *Microbiol. Mol. Biol. Rev.* 76**,** 773-791.

Gomi, S., Majima, K., and Maeda, S. (1999). Sequence analysis of the genome of *Bombyx mori* nucleopolyhedrovirus. *J. Gen. Virol.* 80**,** 1323–1337.

Iyer, L.M., Koonin, E.V., and Aravind, L. (2002). Extensive domain shuffling in transcription regulators of DNA viruses and implications for the origin of fungal APSES transcription factors. *Genome Biol.* 3**,** RESEARCH0012.

Kang, W., Suzuki, M., Zemskov, E.A., Okano, K., and Maeda, S. (1999). Characterization of baculovirus repeated open reading frames (*bro*) in *Bombyx mori* nucleopolyhedrovirus. *J. Virol.* 73**,** 10339-10345.

Pullen, S.S., and Friesen, P.D. (1995). The CAGT motif functions as an initiator element during early transcription of the baculovirus transregulator ie-1. *J. Virol.* 69**,** 3575-3583.

Riedel, H.-D., Heinrich, J., Heisig, A., Choli, T., and Schuster, H. (1993). The antirepressor of phage P1 Isolation and interaction with the C1 repressor of P1 and P7. *FEBS Letters* 334**,** 165-169.

Yarmolinsky, M.B., and Sternberg, N. (1988). "The bacteriophages", in: *Bacteriophage P1.* (ed.) R. Calender. New York: Plenum Press.

Zemskov, E.A., Kang, W., and Maeda, S. (2000). Evidence for nucleic acid binding ability and nucleosome association of *Bombyx mori* nucleopolyhedrovirus BRO proteins. *J. Virol.* 74**,** 6784-6789.

**Legends to the supplementary figures**

**Figure S1. Morphology of *S. pneumoniae* cells after overexpression of pneumococcal toxins.** Cells harbouring **(A)** pLS1ROM-MCS, **(B)** pLS1ROM_Doc, **(C)** pLS1ROM_Ant, **(D)** pLS1ROM_XreA-Ant, **(E)** pLS1ROM_Bro, and **(F)** pLS1ROM_Bro-XreB, were examined after 4 h and 8 h of overexpression. No prominent differences in the morphology of the cells were observed as compared to the wild-type **(A),** even though growth inhibition could be observed when the toxins were overexpressed. Pictures shown were taken at 8 h after overexpression at 100× magnification.

**Figure S2.** **Phylogenetic analyses of PezT homologues *via* neighbour-joining.** Phylogenetic analyses revealed that PezAT formed two clusters: one cluster for PezAT located on PPI-1 and another cluster for PezAT on Tn*5253*.

**Table S1. Presence of PezAT cassettes in the ICEs of genomes of *S. pneumoniae* as found in ICEberg database**

| **Strain** | **Number of PezAT** | **Presence of ICE** |
| --- | --- | --- |
| AP200 | none |  |
| 70585 | 1 | NA* |
| 670-6B | 1 | PPI-1^a^ |
| ATCC 700669 | 2 | one on PPI-1; one on Tn*5253*^b^ |
| BS397 | 1 | NA** |
| BS455 | 1 | NA** |
| BS457 | 1 | NA** |
| BS458 | 1 | NA** |
| CCRI 1974 | none |  |
| CCRI 1974M2 | none |  |
| CDC0288-04 | 1 | NA** |
| CDC1087-00 | none |  |
| CDC1873-00 | 1 | NA** |
| CDC3059-06 | 1 | NA** |
| CGSP14 | 2 | one on Tn*5253*; PPI-1 not found |
| D39 | 1 | PPI-1 |
| G54 | none |  |
| GA04375 | none |  |
| GA17545 | none |  |
| GA17570 | 1 | NA** |
| GA41301 | 1 | NA** |
| GA41317 | none |  |
| GA47368 | 1 | NA** |
| GA47901 | none |  |
| Hungary^19A^-6 | 1 | NA** |
| INV104 | none |  |
| INV200 | 1 | NA* |
| JJA | 1 | PPI-1 |
| MLV-016 | none |  |
| OXC141 | 1 | NA* |
| P1031 | 2 | one on PPI-1; one on Tn*5253* |
| R6 | 1 | PPI-1 |
| SP11-BS70 | none |  |
| SP14-BS292 | 1 | NA** |
| SP14-BS69 | 1 | NA** |
| SP18-BS74 | none |  |
| SP195 | 1 | NA** |
| SP19-BS75 | 1 | NA** |
| SP23-BS72 | 1 | NA** |
| SP3-BS71 | 1 | NA** |
| SP6-BS73 | 1 | NA** |
| SP9-BS68 | 1 | NA** |
| SP-BS293 | 1 | NA** |
| str. Canada MDR 19A | none |  |
| str. Canada MDR 19F | none |  |
| Taiwan19F-14 | none |  |
| TCH8431/19A | none |  |
| TIGR4 | 1 | PPI-1 |
| Total | 34 |  |

NA*: Strain not annotated in ICEberg

NA**: Strain not annotated in ICEberg because the contigs are not assembled

^a^ PPI-1, pneumococcal pathogenicity island 1

^b^ Transposon Tn*5253*
