# Supplementary figures and images for: Functional validation of putative toxin-antitoxin genes from the Gram-positive pathogen Streptococcus pneumoniae: phd-doc is the fourth bona-fide operon
Source: Front Microbiol. 2014 Dec 5;5:677. doi: 10.3389/fmicb.2014.00677 (PMC4257102; doi:10.3389/fmicb.2014.00677)

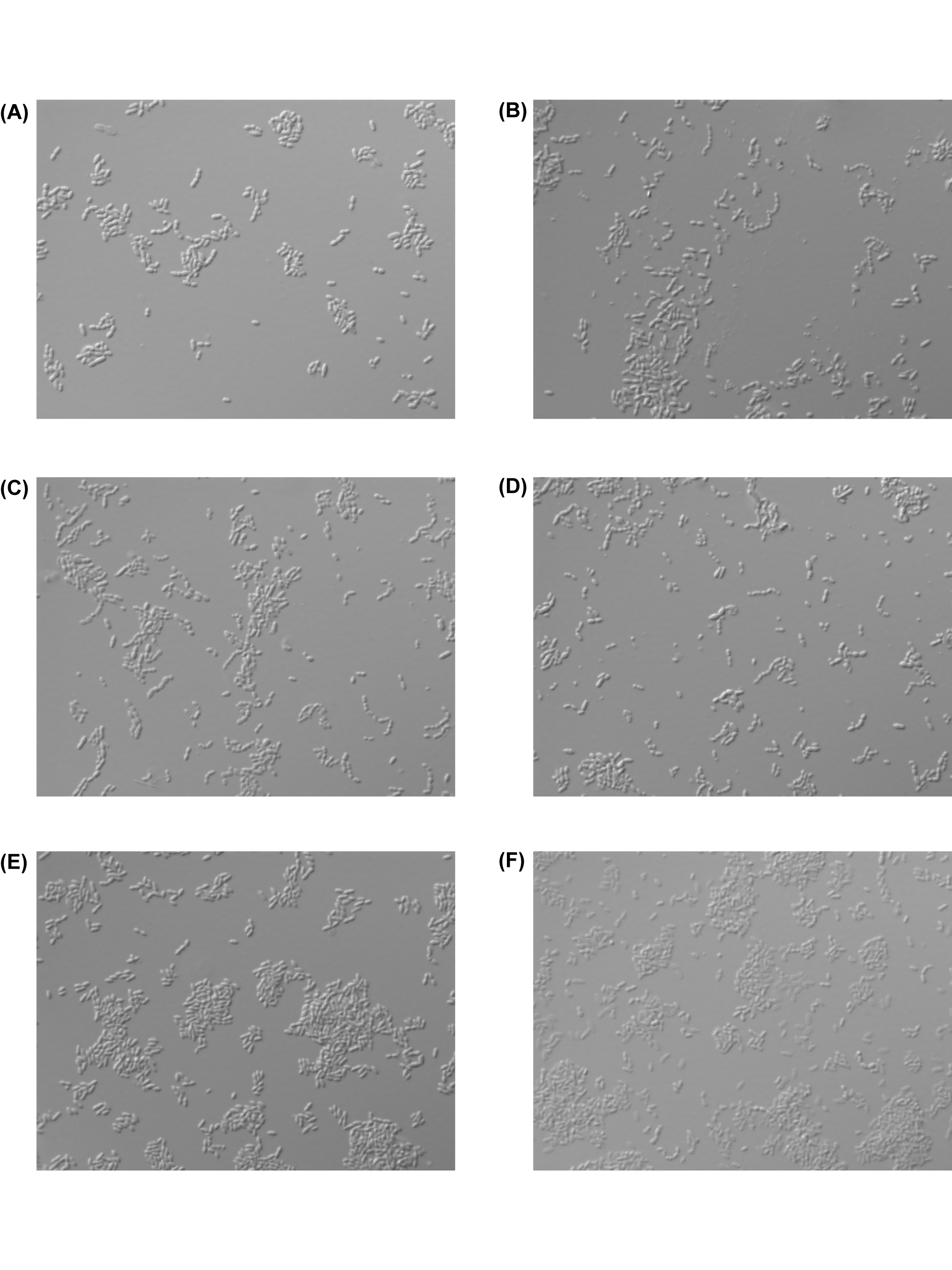

Supplement: Supplementary file 2 [file Image1.TIF]

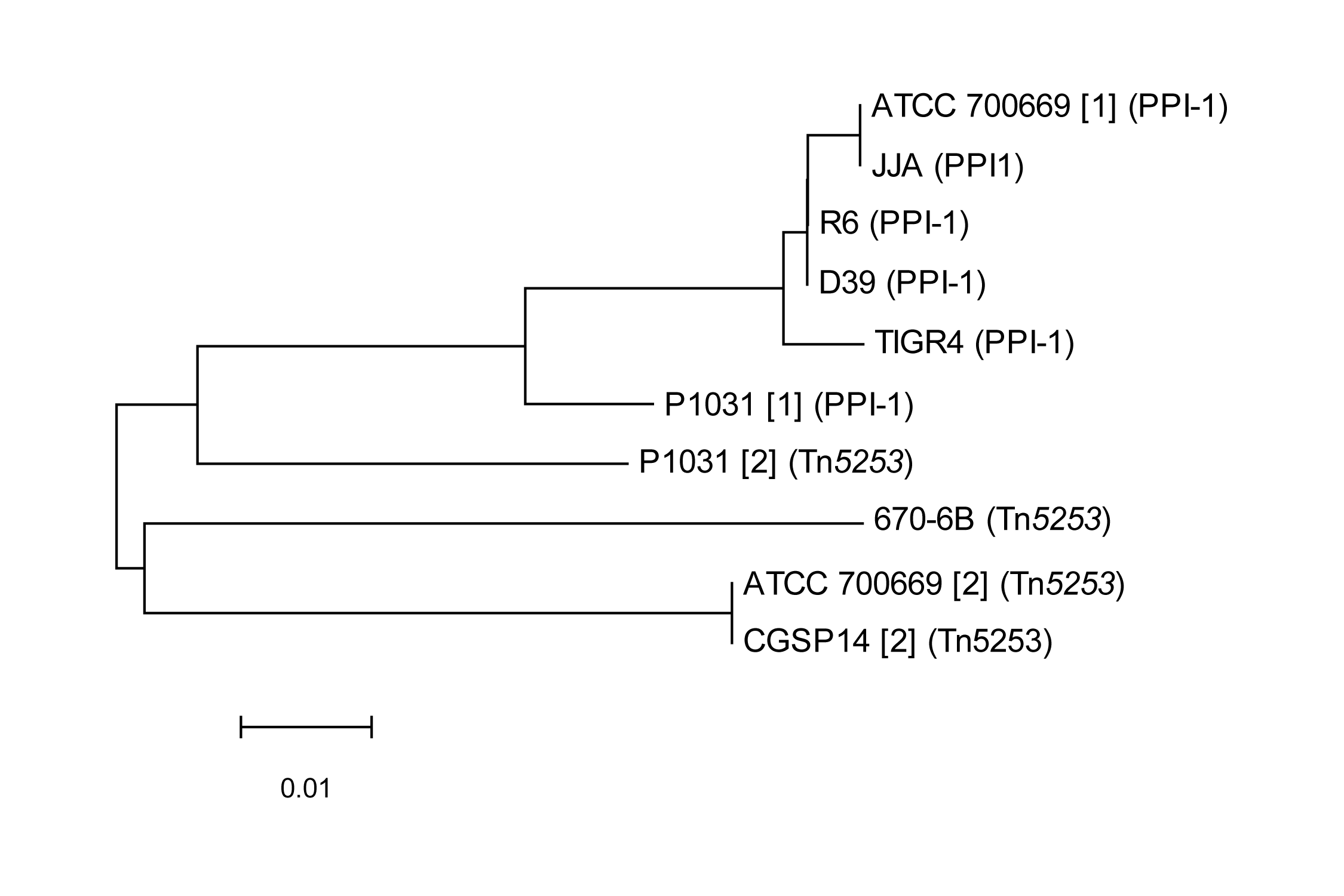

Supplement: Supplementary file 3 [file Image2.TIF]
